# Eye movements reflect capacity-dependent organization of working memory

**DOI:** 10.1371/journal.pbio.3004047

**Published:** 2026-10-13

**Authors:** Qiaoli Huang, Christian F. Doeller

**Affiliations:** 1 Max Planck Institute for Human Cognitive and Brain Sciences, Leipzig, Germany; 2 Kavli Institute for Systems Neuroscience, Norwegian University of Science and Technology, Trondheim, Norway; McGill University, CANADA

## Abstract

Human cognition is capacity-limited, requiring strategies to actively structure information. Eye movements offer a natural mechanism for sequential sampling, but whether such sequences reflect memory organization is unknown. We developed a working-memory task where color-frequency pairings created a latent ordinal structure to optimally reduce memory load. Across two experiments, gaze patterns spontaneously aligned with this structure. Participants sampled items following this sequence during encoding and revisited them during maintenance. Critically, the expression of this structure depended on cognitive demand. In a 3-item task, high performers showed robust sequential sampling during encoding, whereas lower performers compensated with replay-like revisitation during maintenance. Under higher demand (4 items), encoding-based organization was disrupted, and structured revisitation emerged primarily during maintenance. These findings suggest that eye movements provide an overt expression of an internally generated organizational process that flexibly adapts to cognitive demands, revealing a behavioral analogue to neural replay when encoding resources are strained.

## Abstract 2

Human working memory is capacity-limited, requiring information to be actively organized to support efficient encoding and maintenance. This study shows that spontaneous eye movements track latent memory structure and adapt to cognitive demand, offering a noninvasive marker of cognitive load and memory organization.

## Introduction

The human mind operates under strict capacity limits: we cannot process or store every detail of the complex visual world. To optimally deal with these constraints, perception and memory have evolved to rely on efficient coding strategies that exploit the statistical regularities of the environment [1–3]. Real-world scenes are generally structured and predictable, allowing us to use prior knowledge to guide goal-directed behavior. For example, when searching for a book in a library, you do not scan shelves at random; instead, you use the numerical or alphabetical order of catalog labels to predict where the book should be and then move sequentially along the shelves to find it efficiently. Such strategies depend on orderly sampling of structured information, a process that, in vision, is carried out through eye movements.

Because the central fovea offers high acuity but only over a very limited region of the visual field, our eyes must continually move to sample different parts of a scene. This active sensing strategy not only enables efficient visual processing but also provides a unique window into memory systems [4]. Indeed, the oculomotor system is tightly integrated with the brain’s memory circuitry [5], with growing evidence demonstrating that eye movements actively shape memory formation and retrieval. As a result, gaze behavior can serve as a readout of both the accessibility and internal organization of memory contents [6–11], often quantified through summary measures such as the number of fixations or the distribution of saccade vectors.

Building on this perspective, recent research has increasingly focused on the temporal organization of gaze, revealing that eye movements unfold as structured sequences rather than a series of independent fixations. In visually guided tasks, eye-movement trajectories often follow near-optimal policies for maximizing information gain [12–14]. Such sequential sampling provides the visual system with an ordered stream of inputs, which may be especially valuable for constructing stable working-memory representations under capacity constraints. This view aligns with proposals that perceptual and mnemonic systems share efficient-coding principles [15], suggesting that structured eye-movement sequences may reflect active organizational processes during memory formation.

Yet despite these insights, whether and how eye-movement sequences relate to the organization of mnemonic representations in working memory remains unclear. Two questions are particularly critical. First, how are sequential sampling orders established when multiple items must be encoded simultaneously, and to what extent do structured eye movements reflect the organization of concurrent inputs? Second, and more importantly, does this sequence persist or re-emerge during maintenance, when no visual input is available? Demonstrating such internal “replay” of gaze sequences would provide direct evidence that structured eye movements reflect mnemonic organization beyond concurrent visual sampling, paralleling neural replay phenomena thought to support consolidation and mental reorganization [16–20].

To address these questions, we developed a working memory paradigm that embeds a latent structure within the to-be-memorized items. Participants memorized multiple gratings presented simultaneously at distinct spatial locations, each varying in both color (green–blue continuum) and spatial frequency. This dual-feature design imposed substantial working memory demands, encouraging the use of efficient sampling strategies. Critically, spatial frequency was systematically coupled with color hue to create an implicit, ordinal structure across feature space, which could be leveraged to reduce memory uncertainty. This design allowed us to test whether participants would adopt the corresponding structured sampling sequence during encoding and, moreover, whether this sequence would re-emerge during maintenance when stimuli were no longer visible. Finally, by varying memory load across two experiments, we assessed how cognitive demand modulates reliance on encoding-based versus maintenance-based gaze organization, thereby clarifying how oculomotor sequencing relates to the organization of information in working memory.

## Results

### Eye-movement trajectory is modulated by the spatial configuration of the memory contents

Participants were briefly shown three gratings presented simultaneously at distinct spatial locations, each varying in spatial frequency and color. At the end of each trial, they reported the location of a probed feature (either spatial frequency or color) or indicated that the feature was new (Fig 1A). This task challenged working memory capacity by requiring the maintenance of multiple feature–location bindings. To probe potential efficient coding strategies, we introduced an implicit regularity in the stimulus set: lower-frequency gratings were consistently paired with “greener” hues. This contingency established an ordinal structure among the items, providing a potential scaffold for more efficient memory organization. Crucially, on every trial, items could be ranked according to their relative position along this feature gradient (item 1: lowest frequency/greenest; item 2: intermediate; item 3: highest frequency/least green). These ordinally labeled items were assigned to three fixed locations, yielding six possible configuration patterns (Fig 1B). To encourage natural encoding strategies, participants were allowed to move their eyes freely except when a central fixation dot was presented, during which they were required to maintain fixation. Our analyses focused on eye-movement patterns during both the encoding period (when the memory items were visible) and the maintenance period (after their disappearance).

**Fig 1:**
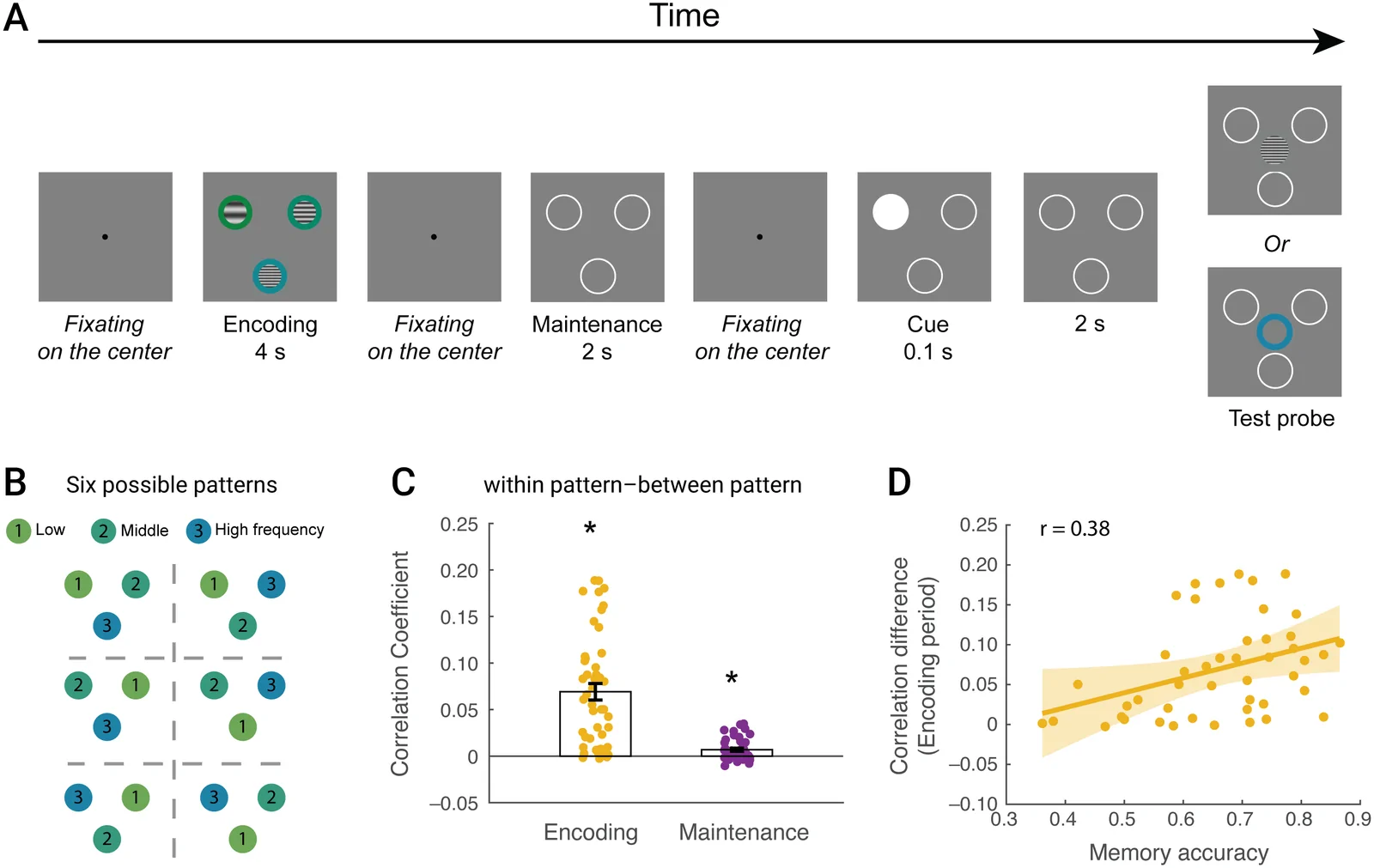
Experimental paradigm and structured gaze trajectory. **(A)** Experimental paradigm (*N* = 46). Participants began each trial by fixating on a central dot. Three gratings (varying in color and spatial frequency) then appeared simultaneously for 4 s, at three fixed, equidistant locations around the center (7° eccentricity). Participants were instructed to memorize each item’s color, frequency, and location. After the gratings disappeared, a central fixation dot reappeared, indicating participants to re-fixate. Next, three white circles occupying the same locations as the gratings were presented for 2 s, followed by another fixation period. Then, the three circles reappeared with one filled for 100 ms to direct attention to that specific location, followed by another 2 s display of all circles. In the end, a test stimulus (color or frequency) appeared at the center of the screen, and either matched one of the memorized items or was novel. Participants needed to report the associated location or indicate “new”. The cue was completely invalid because the test stimulus was equally likely to match the memory item at any given location. Participants could freely move their eyes except during the fixation period. **(B)** Stimulus configuration patterns. There were six possible spatial configuration patterns based on the ordinal rank along the feature gradient. Item 1: lowest frequency, greenest; Item 2: middle frequency, intermediate hue; Item 3: highest frequency, least green. **(C)** Pattern-similarity effect. The contrast of the correlation (mean ± SEM) between within-pattern and between-pattern trial pairs during the encoding period (yellow) and maintenance periods (purple). Each dot indicates individual participants (*: *p* < 0.05). **(D)** Relationship between pattern-similarity effect and memory performance. The scatterplot shows memory accuracy (X-axis) vs. pattern similarity effect in the encoding period (Y-axis). All data and code underlying this figure are publicly available on OSF: https://osf.io/kasr2/.

Behavioral performance was well above chance level (0.25) across all conditions (overall: mean = 0.66, SD = 0.12, *t*_(45)_ = 22.46, *p* < 0.001, Cohen’s *d* = 3.31; color domain: mean = 0.70, SD = 0.13, *t*_(45)_ = 22.87, *p* < 0.001, Cohen’s *d* = 3.37; frequency domain: mean = 0.62, SD = 0.16, *t*_(45)_ = 15.50, *p* < 0.001, Cohen’s *d* = 2.29; color performance is significantly higher: *t*_(45)_ = 3.41, *p* = 0.001, Cohen’s *d* = 0.50). We then asked whether eye-movemen*t* trajectories systematically reflected the embedded cross-feature ordinal structure. Specifically, if participants sampled information based on this latent item order, eye-movement trajectories would be more similar for trials sharing the same configuration (within-pattern) than for trials with different configurations (between-pattern). To test this configuration-specific pattern-similarity effect, we quantified attentional allocation using normalized gaze-to-item distance time courses (Fig 2A), with larger values reflecting stronger relative gaze bias toward a given item. For each item, we computed pairwise correlations between these normalized distance time courses across trials and categorized each pair as within-pattern or between-pattern depending on whether the two trials shared the same configuration or not. Correlations were then averaged across items to yield a single summary measure per participant (see Methods). The difference between within- and between-pattern correlations quantified the pattern-similarity effect, providing an index of structured gaze organization while controlling for baseline biases associated with spatial location.

**Fig 2:**
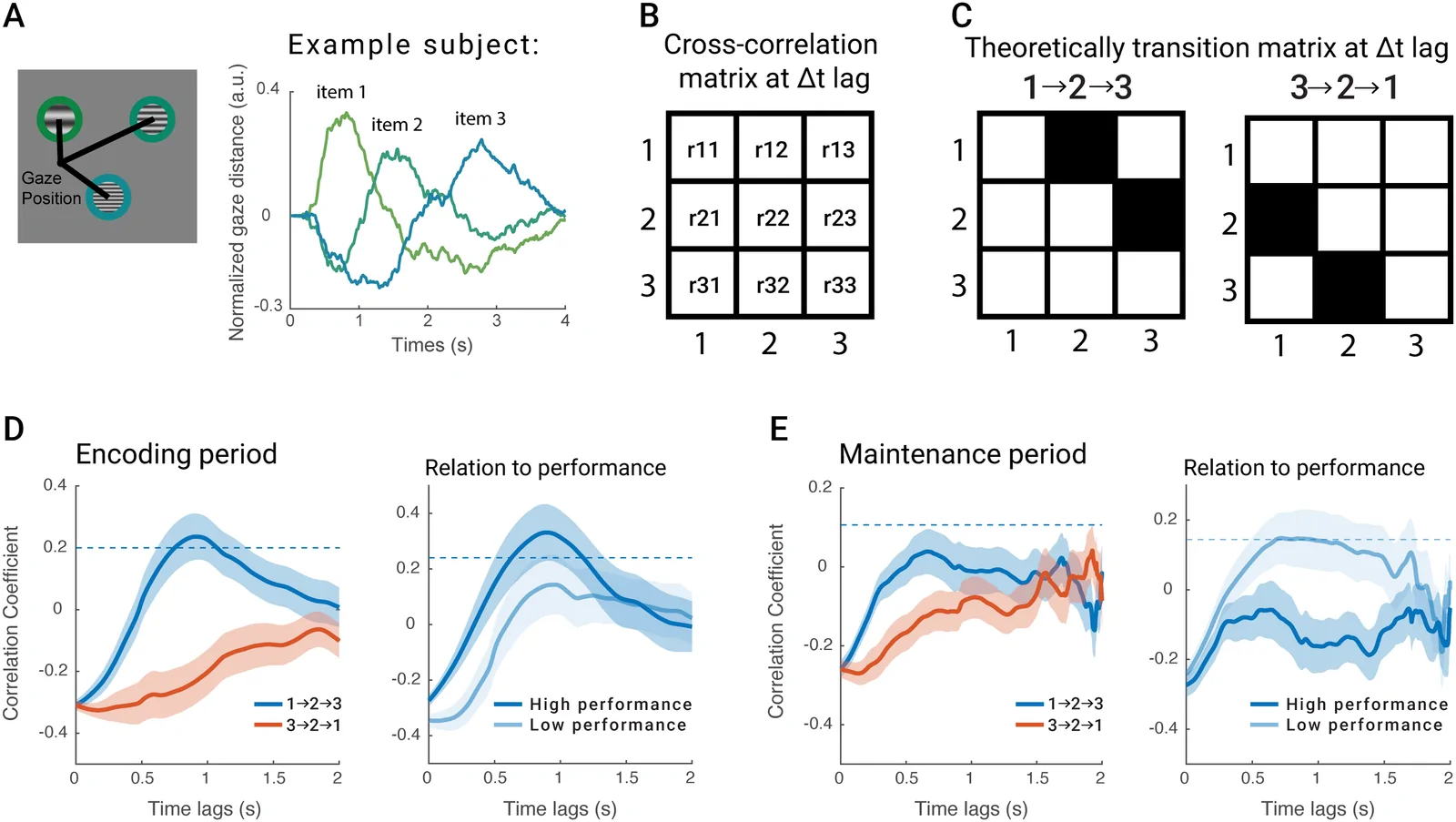
Sequential eye-movement sampling and revisitation. **(A)** Gaze-to-item distance analysis. Left: schematic illustrating the calculation of the gaze distance to each item. Right: time course of normalized gaze distances to individual items (averaged across trials) for a representative participant, with items labeled by their ordinal rank. **(B)** Empirical gaze cross-correlation matrix. Each element represents the cross-correlation between the gaze-bias time course of one item and the temporally lagged gaze-bias time course of another item at a given temporal lag. **(C)** Theoretically transition model: 1 → 2 → 3 sequence (left panel); 3 → 2 → 1 sequence (right panel). **(D)** Sequence strength during encoding. Left: strength of 1 → 2 → 3 (blue) and 3 → 2 → 1 (red) sequences, separately quantified by the correlation between empirical cross-correlation matrix in (B) and each theoretical transition model in (C) at each temporal lag. Right: 1 → 2 → 3 sequence strength in high (*N* = 23, dark blue) and low-performance (*N* = 23, light blue) groups. The dashed line indicates the statistical threshold (*p* < 0.05) derived from permutation testing and corrected for multiple comparisons across temporal lags. **(E)** Sequence strength during maintenance. The same analysis as in **(D)**, applied to the maintenance period. All data and code underlying this figure are publicly available on OSF: https://osf.io/kasr2/.

As predicted, we observed a significant pattern-similarity effect, i.e., gaze trajectories were more similar within-pattern than between-pattern during both the encoding (*t*_(45)_ = 7.77, *p* < 0.001, Cohen’s *d* = 1.15) and maintenance period (*t*_(45)_ = 4.15, *p* < 0.001, Cohen’s *d* = 0.61, Fig 1C). This effec*t* remained when we balanced trial numbers across within- and between-pairs (see S1A Fig) and was already presented in the first half of the trials (see S4 Fig). Critically, participants who showed a stronger pattern-similarity effect during encoding also exhibited higher memory accuracy (*r* = 0.38, *p* = 0.009, Fig 1D). This suggests that stronger configuration-specific organization of gaze during encoding reflects more structured mnemonic processing associated with better memory performance.

### Sequential eye movements reveal performance-dependent sampling and revisitation

Having established that gaze patterns reflect structured mnemonic processing, we next examined the temporal organization of these trajectories. Specifically, we asked whether participants sampled items in a systematic sequence that strictly followed their ordinal structure (either 1 → 2 → 3 or 3 → 2 → 1, where 1–3 correspond to items ordered from lowest to highest spatial frequency). To quantify sequential structure, we applied a sequenceness analysis framework [21]. We first computed cross-correlation coefficients between all pairs of item-specific gaze-bias time courses to generate a correlation matrix for each temporal lag (Fig 2B). Next, we specifically tested two transition models against these empirical matrices: rank-increasing sequence 1 → 2 → 3 model which contained transitions from item 1 to item 2 and from item 2 to item 3, and a reverse 3 → 2 → 1 model (Fig 2C, see Methods). Permutation tests with item-label shuffling showed that only the 1 → 2 → 3 sequence significantly explained the empirical data, with a prominent effect at lags of 0.75–1.07 s (peak = 0.91 s, Fig 2D, left). To assess behavioral relevance, we split participants into high- and low-performance groups based on a median split of memory accuracy (*N* = 23 per group). Strikingly, the high-performance group exhibited a robust 1 → 2 → 3 sequential profile (lags: 0.63–1.17 s; peak = 0.89 s), whereas the low-performance group did not (Fig 2D, right), indicating that ordered gaze sampling during encoding was more strongly expressed in participants with better memory performance. This sequential pattern also became more strongly expressed with experience across trials (see S4 Fig) and remained visible when looking at discrete fixation sequence (see S7I Fig).

Having characterized the sequential structure during encoding, we turned to the maintenance period to determine whether the same sequence re-emerged after the stimuli were no longer visible. To address this, we applied the same sequenceness analysis to the maintenance period. At the group level, no significant sequential eye-movement pattern was observed. However, analysis of individual differences revealed a striking and unexpected dissociation: contrary to what might be assumed, the low-performance group exhibited significant sequential “revisitation” at lags of 0.68–0.95 s (peak: 0.72 s), whereas the high-performance group showed no evidence. Importantly, control analyses confirmed this effect was not explained by spatial biases, as sequence analysis based solely on item locations yielded no significant structure (S2 Fig).

Taken together, these findings show that structured eye-movement patterns were closely associated with memory performance and differed between encoding and maintenance as a function of individual ability. High-performing participants showed robust ordinal sampling (lowest → highest) during encoding but no evidence of sequential revisitation during maintenance, consistent with the idea that efficient initial encoding reduces the need for subsequent stabilization. In contrast, low-performing participants failed to deploy this encoding strategy and instead exhibited revisitation during maintenance, consistent with post-encoding reorganization and stabilization of memory traces. This behavioral pattern aligns with evidence from neural replay, where replay primarily supports the consolidation of weakly encoded information, whereas strong initial encoding diminishes the need for later reactivation [22,23].

### Retro-cue initiates sequential gaze transitions following the ordinal structure

To further validate the observed sequential organization, we examined eye-movement sequence following a retro-cue presented immediately after the maintenance period. The retro-cue was designed to exogenously direct attention and gaze. If effective, the initial saccade should be directed toward the cued item, whereas subsequent saccades may reveal the internal priority structure of the remaining memory representations. Given the observed 1 → 2 → 3 ordering, we predicted that: (1) when item 1 was cued, the second saccade would be more likely to target item 2 than item 3, and (2) when item 2 was cued, the second saccade would be more likely to target item 3 than item 1. We first verified the effectiveness of the retro-cue by analyzing first-saccade destinations (Fig 3A). Fixation heatmaps following cue onset revealed a strong spatial bias toward the cued locations (Fig 3B). Quantitatively, first saccades were directed toward the cued location significantly more often than expected by chance (1/3; *t*_(45)_ = 7.19, *p* < 0.001, Cohen’s *d* = 1.06; Fig 3C).

**Fig 3:**
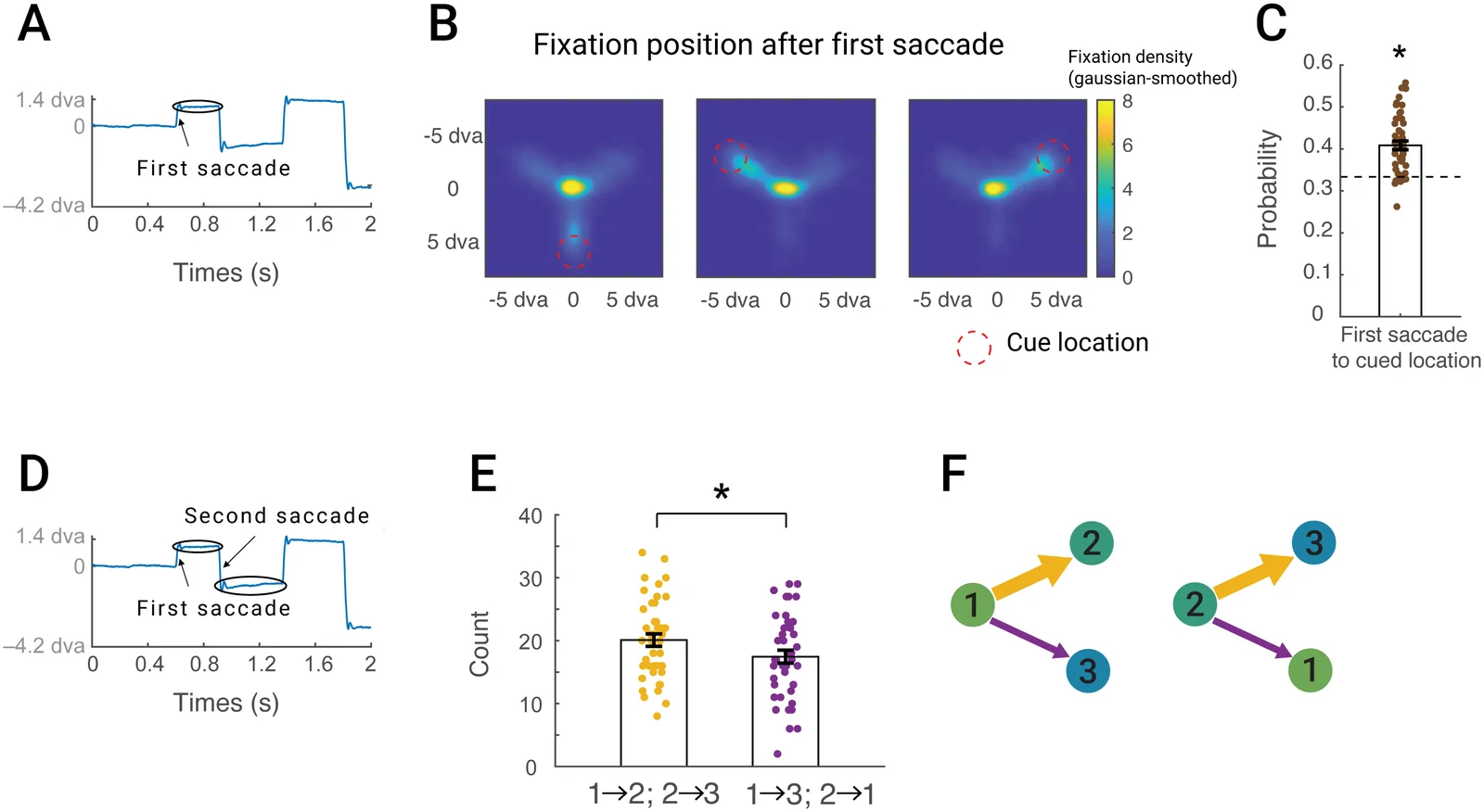
Ordered eye-movement transition following the retro-cue. **(A)** Example gaze trajectory (y-axis position over time) illustrating the first saccade after retro-cue onset. **(B)** First-saccade destination heatmaps for each cue location (bottom, upper left, upper right). Red dashed circles indicate the cued locations. Note that here we intended to capture which item location the first saccade landed closest to, rather than requiring the saccade to land precisely on a specific location. **(C)** Proportion of the first saccade directed toward the cued location. Each dot indicates an individual participant (*: *p* < 0.05). Dashed line indicates the chance level (1/3). **(D)** Example gaze trajectory illustrating the second saccade following the retro-cue. **(E)** Second-saccade transition frequencies: left bar (mean ± SEM) shows predicted sequential transitions (1 → 2, 2 → 3); right bar (mean ± SEM) shows alternatives (1 → 3, 2 → 1). Each dot indicates an individual participant (*: *p* < 0.05). **(F)** Schematic illustrating the 1 → 2 → 3 sequence. Preferred transitions (large arrows) occur more frequently than non-preferred transitions (small arrows): after item 1 was cued, gaze is more likely to move to item 2 than item 3; after item 2 was cued, it is more likely to move to item 3 than to item 1. All data and code underlying this figure are publicly available on OSF: https://osf.io/kasr2/.

To test whether the second saccade (Fig 3D) followed the predicted sequential order, we focused on trials that met three criteria: (1) the first saccade landed on the cued location, confirming effective cueing; (2) either item 1 or item 2 (the positions from which ordered transitions are interpretable) was cued; and (3) at least two distinct saccades were made. Note that 4 participants were excluded from further analyses because fewer than 20 trials satisfied these requirements. Consistent with our hypothesis, second-saccade transitions exhibited strong directionality: item 1 → item 2 and item 2 → item 3 transitions occurred significantly more frequently than alternative transitions (item 1 → item 3 and item 2 → item 1) (*t*_(41)_ = 3.33, *p* = 0.002, Cohen’s *d* = 0.51; Fig 3E), which robustly validated the optimal 1 → 2 → 3 sequential organization. Additional analyses including all six possible transitions (1 → 2, 1 → 3, 2 → 1, 2 → 3, 3 → 1, 3 → 2) yielded a similar pattern. Forward transitions (1 → 2, 2 → 3) occurred significantly more often than backward transitions (2 → 1, 3 → 2) (*t*_(41)_ = 2.36, *p* = 0.04, Cohen’s *d* = 0.32) and long-distance transition (1 → 3, 3 → 1) (*t*_(41)_ = 3.35, *p* = 0.004, Cohen’s *d* = 0.46) (S5B Fig). Together, these results show that post-maintenance gaze transitions precisely mirror the same ordinal structure observed during encoding/maintenance, confirming the active sequential organization of memory representation.

### Central-fixation constraint degraded memory performance

The association between structured gaze and memory performance in Experiment 1 raised an important question: what happens to memory organization when these natural eye-movement patterns are restricted? To address this question, we conducted a control experiment using the same 3-item task as in Experiment 1, except that participants were required to maintain central fixation during encoding and maintenance (Fig 4A). Compared with the free-viewing condition, memory accuracy (mean = 0.55, SD = 0.09) was lower under this fixation constraint (Fig 4B, *t*_(75)_ = 4.37, *p* < 0.001, Cohen’s *d* = 1.01). Moreover, the configuration-specific pattern-similarity effect observed under free viewing was no longer significant under the fixation constraint (Fig 4C, encoding period: *t*_(30)_ = 1.44, *p* = 0.16, Cohen’s d = 0.26; main*t*enance period: *t*_(30)_ = –0.43, *p* = 0.67, Cohen’s *d* = –0.08), nor was *t*he corresponding correlation with memory performance (Fig 4D, *r* = 0.27, *p* = 0.15). Together, these findings are consistent with the view that overt eye movements reflect organization of memory representations.

**Fig 4:**
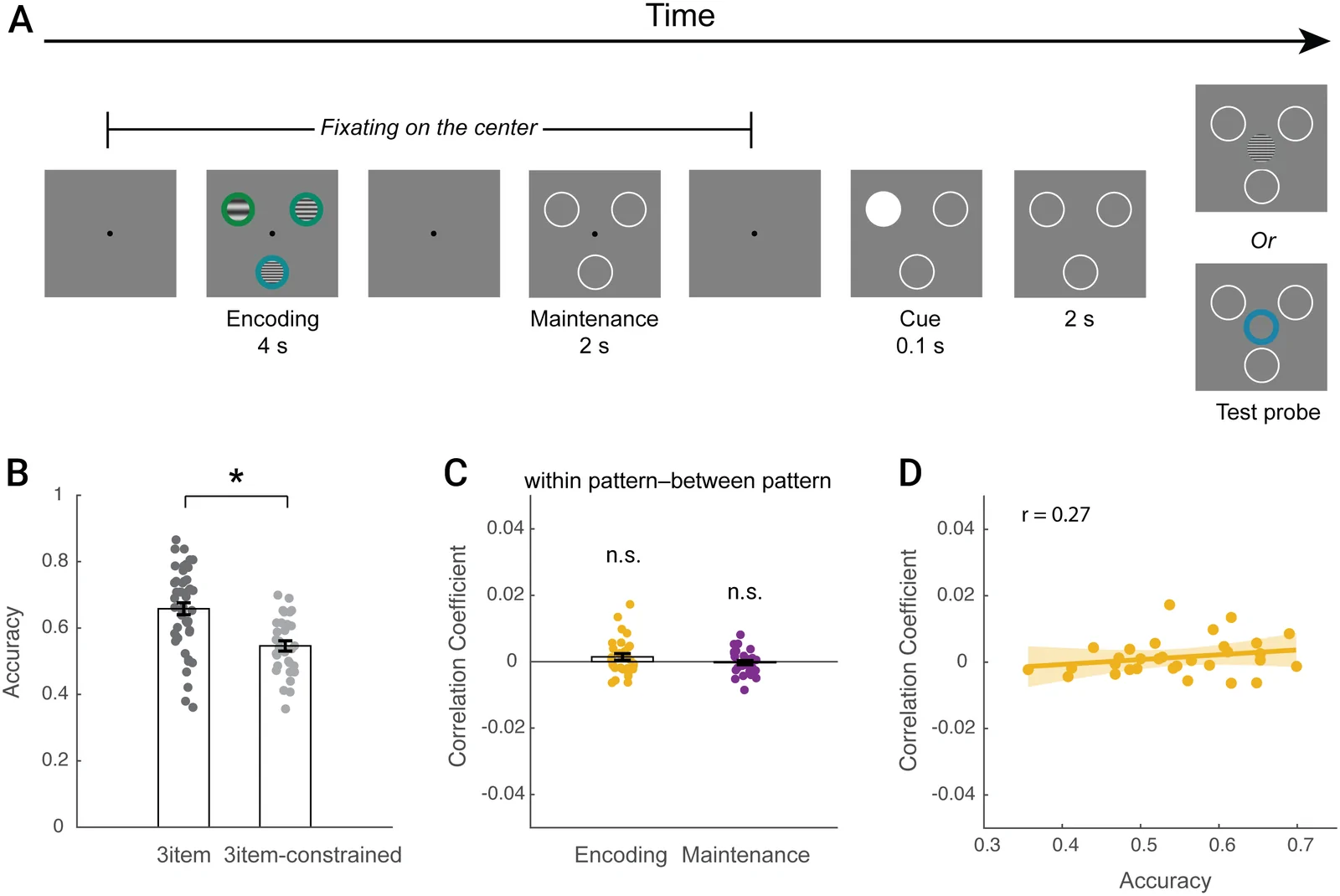
Central-fixation control experiment: paradigm and results. **(A)** Experimental paradigm (*N* = 31). The task was identical to Experiment 1, except that participants had to fixate at the center of the screen during both encoding and maintenance periods. **(B)** Pattern-similarity effect. Bars show the mean difference between within-pattern and between-pattern trial-pair correlations during encoding (yellow) and maintenance (purple), with error bars indicating SEM. Dots represent individual participants (*: *p* < 0.05). **(D)** Relationship between pattern similarity and memory performance. The scatterplot shows memory accuracy (X-axis) as a function of the pattern-similarity effect during the encoding period (Y-axis). All data and code underlying this figure are publicly available on OSF: https://osf.io/kasr2/.

### High cognitive load shifts structured organization from encoding to maintenance

Our previous results suggested that structured gaze sequences are closely linked to successful memory organization, but that their expression may depend on available cognitive resources. High performers showed robust sequential sampling during encoding, whereas low performers appeared to shift this organizational process into the maintenance period, where sequential revisitation may reflect post-encoding reorganization of less robustly encoded information. This pattern raised a key question: Does increasing cognitive load disrupt the expression of sequential organization during encoding and promote a compensatory shift toward structured revisitation during maintenance? To address this, we designed a higher-load 4-item memory task (Fig 5A), analogous to the 3-item paradigm but with one additional item to remember.

**Fig 5:**
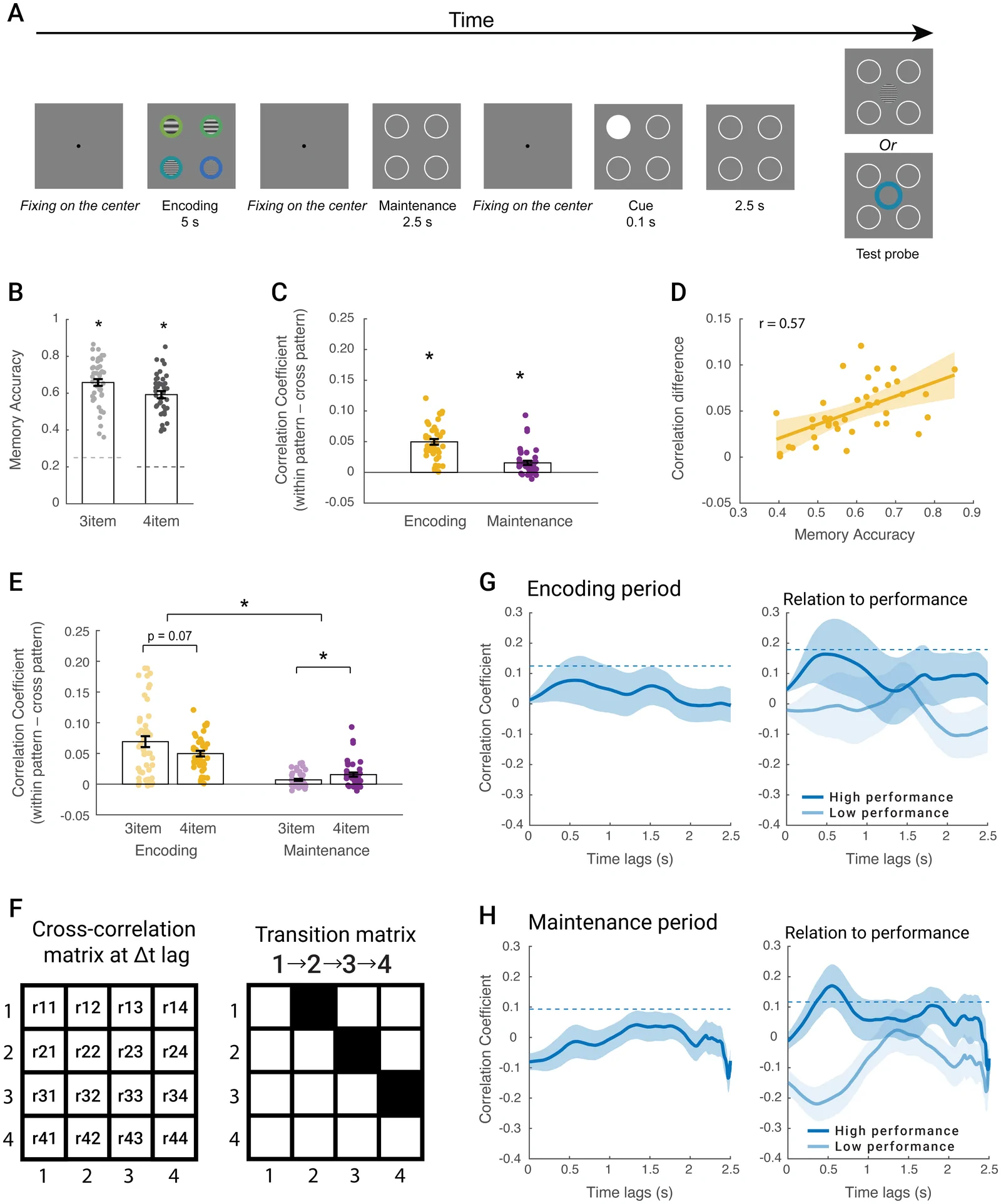
Experimental paradigm and results of 4-item task. **(A)** Experimental paradigm for the 4-item memory task (*N* = 40). The design was identical to the 3-item task, except that participants were asked to remember four items. **(B)** Comparison of memory accuracy (mean ± SEM) between 3-item (gray) and 4-item (black) tasks. Each dot indicates one participant (*: *p* < 0.05). **(C)** Pattern-similarity effect. Difference in correlation (mean ± SEM) between within-pattern and between-pattern trial pairs, shown separately for the encoding period (yellow) and maintenance period (purple). **(D)** Relationship between memory performance and pattern similarity. Scatterplot showing that participants with stronger pattern-similarity effects during encoding exhibited higher memory accuracy. **(E)** Comparison of pattern similarity effects (mean ± SEM) between 3-item (light color) and 4-item (dark color) tasks for both memory encoding (yellow) and maintenance (purple) periods. **(F)** Left: empirical correlation matrix at Δ*t* lag formed by *t*he pairwise cross-correlation between items. Right: theoretical transition matrix representing the 1 → 2 → 3 → 4 sequence. **(G**) Sequential organization during encoding. Left: time course of 1 → 2 → 3 → 4 sequence strength (mean ± SEM) across all participants during the encoding period. Right: sequence strength in high (*N* = 20, dark blue) and low-performance (*N* = 20, light blue) groups. Dashed lines indicate permutation-derived significance thresholds corrected for multiple comparisons. **(H)** Sequential organization during maintenance. Same as (G) but showing 1 → 2 → 3 → 4 sequence strength during the maintenance period. All data and code underlying this figure are publicly available on OSF: https://osf.io/kasr2/.

Participants performed well above chance (0.2; overall: mean = 0.59, SD = 0.11, *t*_(39)_ = 22.33, *p* < 0.001, Cohen’s *d* = 3.53 (Fig 5B right); color domain: mean = 0.61, SD = 0.12, *t*_39)_ = 21.05, *p* < 0.001, Cohen’s *d* = 3.33; frequency domain: mean = 0.57, SD = 0.14, *t*_(39)_ = 17.15, *p* < 0.001, Cohen’s *d* = 2.71; performance in color domain is marginally higher than in *t*he frequency domain: *t*(39) = 1.73, *p* = 0.09, Cohen’s *d* = 0.27). Directly comparing the memory accuracy be*t*ween the 3-item and 4-item tasks was not appropriate because they differed in chance level (0.25 versus 0.2). We therefore compared performance using chance-corrected accuracy, $\frac{\left(p-c\right)}{\left(\text{1}-c\right)}$, where p is observed accuracy and c is the chance level. Under this normalization, performance in the 4-item task (mean = 0.49, SD = 0.14) was lower than in the 3-item task (mean = 0.54, SD = 0.16) (*t*_(84)_ = 1.62, *p* = 0.05, one-tailed, Cohen’s *d* = 0.35), although the statistical evidence for this difference was modest. Nevertheless, the 4-item task imposed greater cognitive demands by design, as par*t*icipants were required to maintain one additional item in working memory. The chance-corrected behavioral comparison provides convergent, albeit modest, support for this increased difficulty.

Replicating our 3-item analysis, we first examined whether eye-movement trajectories also showed a pattern-similarity effect, i.e., greater similarity for trials sharing the same configuration pattern relative to trials with different patterns. This pattern-similarity effect was again significant during both encoding (*t*_(39)_ = 10.66, *p* < 0.001, Cohen’s *d* = 1.69) and maintenance (*t*_(39)_ = 4.54, *p* < 0.001, Cohen’s *d* = 0.72, Fig 5C), wi*t*h the encoding effect strongly associated with memory accuracy (*r* = 0.57, *p* < 0.001, Fig 5D). Critically, comparison of the 3-item and 4-item tasks revealed a significant interaction effect (Fig 5E, *F*_(1,84)_ = 6.84, *p* = 0.01, *η*_*p*_^2^ = 0.08): compared with the 3-item task, the 4-item task showed a marginally reduced pattern-similarity effect during encoding (*t*_(84)_ = −1.86, *p* = 0.07, Cohen’s *d* = 0.40) but a significantly s*t*ronger effect during maintenance (*t*_(84)_ = 2.35, *p* = 0.02, Cohen’s *d* = 0.51). These findings suggest tha*t* increasing task demand was associated with a shift in configuration-specific gaze organization across processing stages, with relatively weaker expression during encoding but stronger expression during maintenance.

We next asked whether the more fine-grained sequential structure of eye movements showed a comparable shift. Following the analysis in the 3-item task, we constructed 4 × 4 time-lagged cross-correlation matrices and test whether they reflected the 1 → 2 → 3 → 4 sequence (Fig 5F). During encoding, no significant group-level expression of this sequential pattern was observed (Fig 5G). Although this may appear inconsistent with the encoding-period pattern-similarity effect, the two analyses address different levels of structure. The pattern-similarity analysis asks whether participants exhibit similar configuration-specific gaze patterns across trials, whereas the sequenceness analysis additionally requires these gaze patterns to follow a stable ordinal trajectory over time.

We then again divided participants into high- and low-performance groups based on a median split of memory accuracy (*N* = 20 per group). Although high-performing participants showed a trend toward structured sampling during encoding, this effect did not reach statistical significance (see S3 Fig for nonsignificant 4 → 3 → 2 → 1 sequence). In contrast, during maintenance (Fig 5H), although no significant sequence was observed at the group level, high performers exhibited significant sequential revisitation at lags of 0.35–0.74 s (peak at 0.55 s). This result resembled the maintenance-period revisitation pattern observed in lower performers in the 3-item task, consistent with the idea that increasing cognitive demand shifts the expression of sequential organization toward maintenance, where internally generated revisitation may reflect compensatory reorganization of structured memory representations.

Taken together, our experiments demonstrate that eye movements reflect adaptive memory organization according to the latent ordinal structure. Critically, the deployment of this organizational strategy appears to depend on available cognitive resources. In the 3-item task, where encoding demands were relatively lower, high performers engaged in structured sampling during encoding, whereas low performers exhibited sequential revisitation during maintenance, consistent with compensatory post-encoding reorganization. In the more demanding 4-item task, encoding-phase sequential organization was less reliably expressed, while high performers developed structured revisitation during maintenance. Thus, under higher cognitive load, sequential organization appears to shift toward the maintenance period, potentially providing an opportunity to reorganize less robustly encoded information. More broadly, these findings reveal sequential eye movements as an overt expression of an active, adaptive mechanism for internally organizing information in working memory.

## Discussion

The fovea’s high acuity comes at the cost of limited spatial coverage, necessitating frequent eye movements to sample the visual input. Working memory faces a similar constraint: its limited capacity requires efficient strategies for organizing information during encoding and retention [24,25]. To investigate how eye movements reflect such organization under demanding conditions, we asked participants to remember three simultaneously presented gratings that varied in color (from green to blue) and spatial frequency. This design largely challenged working memory capacity by requiring the maintenance of multiple feature–location bindings. Critically, lower spatial frequencies were consistently paired with “greener” hues, creating a latent ordinal structure that afforded an ordered sampling sequence—either ascending or descending in feature space—that could help to reduce memory load. Across experiments, eye-movement trajectories reflect structured mnemonic processing and were closely associated with memory performance. High-performing participants expressed this structured sequence during encoding, whereas low-performing participants only exhibited sequential revisitation during maintenance, suggesting that the timing of sequential organization may depend on available cognitive resources. Supporting this interpretation, increasing memory demand in the 4-item task produced a similar shift, with sequential organization becoming less evident during encoding and emerging more prominently during maintenance. Together, these findings show that eye movements do not merely accompany memory but provide an overt expression of an internally generated, adaptive organizational process that shifts from structured sampling during encoding toward sequential revisitation during maintenance as cognitive demands increase.

To further examine the functional relevance of overt eye movements to memory performance, we conducted a control experiment in which participants performed the same 3-item task while maintaining central fixation during both encoding and maintenance. Compared with the free-viewing condition, the fixation constraint was associated with reduced memory performance, the disappearance of the configuration-specific pattern-similarity effect, and the loss of the relationship between gaze structure and memory performance. These findings indicate that allowing natural eye movements is associated with better memory performance and the expression of structured gaze patterns in this task. However, they should be interpreted cautiously because maintaining central fixation also limits foveal sampling of peripheral stimuli. Thus, the present manipulation cannot determine whether the performance reduction reflects impaired visual information acquisition, disruption of memory-related organization, or both, and therefore does not establish that eye movements themselves structure memory representations. Together with the free-viewing experiments, these results support the interpretation that structured eye movements provide an overt expression of an internally generated organizational process, while the present data do not establish that eye movements themselves are necessary for, or causally create, this organization. Importantly, our focus on overt eye movements does not imply that they constitute the sole mechanism underlying memory organization. Covert attentional processes may also organize memory representations in a sequential manner. Future studies combining eye-movement recordings with neural measures, such as EEG/MEG, will be important for dissociating the respective contributions of overt eye movements and covert sequential attention to memory organization and for clarifying how internally generated neural sequences relate to eye-movement behavior.

Our key finding reveals that eye-movement strategies are dynamically reorganized depending on task demands. Under low demands, participants exhibited an ordered sequential sampling pattern during encoding. As demands increased, however, this stable sequential strategy was no longer reliably expressed during encoding and instead re-emerged during the maintenance period, suggesting a shift toward post-encoding organization of memory representations. Importantly, this does not imply that the latent structure failed to be acquired during encoding. Rather, together with the preserved pattern-similarity effect, the findings suggest that the latent structure was still represented, but its overt sequential expression became less stable under higher memory load. The maintenance-period sequence may therefore reflect post-encoding reorganization or reinforcement of this latent structure. This interpretation parallels neural replay literature, where weakly encoded information elicits stronger offline reactivation [22,23], and aligns with eye-tracking evidence showing that stable or well-learned memories require less exploratory sampling [6,26], whereas increased cognitive load leads to stronger fixation reinstatement [27]. Together, these results suggest that when encoding demands exceed cognitive capacity, the brain compensates by developing sequential resampling during the maintenance period (despite the absence of visual input) as an active process for reorganizing and reinforcing less robustly encoded memory representations.

Although these findings converge on a load-dependent shift from encoding toward maintenance, this interpretation should be considered in light of differences in sensitivity between the two experiments. The 4-item task contained a smaller number of trials per configuration, which could reduce sensitivity for detecting configuration-specific and sequential gaze structure, alongside other differences inherent to the 3-item and 4-item designs. However, our trial-matched analysis showed that subsampling the 3-item experiment to the same number of trials per configuration as the 4-item experiment reproduced the overall pattern observed in the full dataset, suggesting that trial number alone is unlikely to account for the observed difference. Nevertheless, future studies using more closely matched designs will be important for determining how sequential gaze organization generalizes across different memory loads.

Our previous work using EEG/MEG decoding has shown that spontaneous neural replay occurs during working memory maintenance and retrieval, and that this replay supports behavioral performance [17,28–30]. Building on these findings, the present study uses eye movements as a high-resolution behavioral readout of replay-like processes and demonstrates that the expression of these sequential processes is flexibly modulated by task demands (difficulty). Notably, if greater task difficulty is considered analogous to an earlier stage of learning, these findings point to a strategic shift: early in learning (or under high demands), participants may rely more strongly on the maintenance period to reorganize information, whereas later in learning (or under low demands), they can apply well-established encoding strategies directly and efficiently during stimulus presentation. This progression closely mirrors skill acquisition, in which novices require extensive post-hoc rehearsal while experts implement efficient routines online.

Unlike traditional neural replay paradigms using explicitly ordered sequences [16–20], our design presented all items simultaneously, requiring participants to self-organize a sampling order. Despite this, eye trajectories consistently followed an ordered path through feature space (e.g., low-to-high frequency), indicating internally generated organization. This aligns with evidence from decision-making and visual search tasks [12–14], where gaze trajectories reflect planned, efficiency-driven sampling. Moreover, the consistent tendency to initiate sequences with low-frequency items may reflect an anchoring strategy, whereby one end of the ordinal structure serves as a starting point for sequential sampling. However, the present data do not establish the mechanism underlying this initial bias. Although such an account could potentially relate to broader theories of ordered magnitude representation [31,32], this interpretation remains speculative.

An alternative explanation is that the observed sampling sequences were driven, at least in part, by low-level visual salience [33] rather than strategic memory organization. This possibility is plausible because spatial-frequency content has been shown to influence gaze allocation [34]. If the items with different spatial frequencies within a display differed systematically in visual salience, participants might initially orient toward the most salient item and subsequently move toward the next most salient item, thereby generating an ordered sampling sequence. However, there is no clear evidence for a simple monotonic relationship between spatial frequency and gaze allocation or visual salience. In our task, ordinal position was defined by the relative spatial frequencies within each trial rather than by fixed absolute spatial-frequency values. Importantly, we did not independently quantify or manipulate the salience of the individual gratings and therefore cannot determine whether the low-, medium-, and high-frequency items within a trial formed a corresponding salience gradient. Thus, bottom-up salience and strategic organization provide alternative, and potentially complementary, explanations for the sequential sampling observed during encoding that cannot be fully distinguished by the present data.

Importantly, our broader interpretation does not require the encoding sequence to arise exclusively from top-down organization. Structured gaze was also expressed during maintenance, when the stimuli were no longer visible and therefore could not exert a concurrent bottom-up influence on gaze. Moreover, the expression of structured gaze differed across task stages and memory demands, with weaker encoding-related organization but stronger maintenance-related organization under higher task demand. Thus, even if bottom-up salience contributed to establishing the initial sampling sequence during encoding, it cannot by itself account for the expression of structured gaze in the absence of concurrent visual input during maintenance. We therefore interpret the overall pattern as consistent with an interaction between perceptual sampling and internally generated memory organization: bottom-up salience may contribute to establishing the sampling sequence during encoding, whereas structured gaze during maintenance reflects organization expressed in the absence of concurrent visual input. Future studies that directly manipulate or independently quantify stimulus salience will be important for determining their respective contributions.

Over the past decades, eye-tracking has emerged as a powerful tool for probing memory-related cognition [4,5]. One particularly striking insight is how rapidly memory expression is reflected in eye movements. Early work established this link by analyzing summary statistics, such as visual sampling duration and saccade vector distributions, which reliably predict memory performance and hippocampal engagement [6,7,35–39]. Later studies used scanpath analysis to further demonstrate that during retrieval, individuals’ gaze patterns precisely reinstated those from the encoding phase [40–43]. At the same time, a broader literature has increasingly emphasized that visual working memory is closely linked to oculomotor system, with bidirectional influences between internal representations, attentional prioritization, planning and execution [44–53]. Extending this work, our study characterizes the fine-grained temporal architecture of sequential eye movements during working memory. The results show that even in the absence of external input, gaze reflects an active process of internally generated revisitation through which multiple memory representations are sequentially organized. The sequential “revisitation” observed here is closely related to gaze refixation phenomena reported in the eye-movement literature [54–56], but we use this term to emphasize its potential role in memory organization during the maintenance period, after the stimuli have disappeared from the screen.

Our finding that gaze behavior was related to working memory performance differs somewhat from previous studies in which spatial gaze biases during working memory tasks were primarily interpreted as indices of internal attentional selection rather than as direct predictors of behavioral success [9,57]. One possible explanation is that the two paradigms capture different aspects of memory processing. Previous studies focused on gaze biases toward a single prioritized memorized location, whereas the present study examined whether gaze followed a structured sequential sampling pattern across multiple items embedded within a latent ordinal structure. We therefore think it is plausible that the relationship with memory performance emerged because the measured gaze behavior reflected how multiple memory representations were organized or reorganized over time, rather than simply which individual representation was momentarily prioritized. In this sense, gaze biases toward a remembered location may primarily serve as an index of internal attentional selection, whereas the sequential gaze patterns observed here may reflect the organization of multiple representations in working memory. Rather than being contradictory, these findings suggest that eye movements can reveal dinstict aspects of working-memory processing, ranging from the selection of individual representations to the sequential organization of relational memory structure across multiple representations.

In conclusion, we show that the expression of structured eye-movement strategies shifts with increasing cognitive demands, from sequential sampling during encoding toward structured revisitation during maintenance. Together with the fixation-control experiment, these findings suggest that eye movements provide an overt expression of dynamic memory organization that flexibly changes with task demands. By capturing these dynamics in real time, our study positions gaze as a window onto adaptive cognitive organization, offering a powerful noninvasive marker of cognitive load, memory organization, and internal sampling in complex environments.

## Methods

### Participants

One hundred twenty-seven participants (aged 18–35 years) took part in the study: 48 in Experiment 1 (3-item memory), 38 in the central-fixation control experiment, and 41 in Experiment 2 (4-item memory). They were recruited via the internal database recruitment system of Max Planck Institute for Human Cognitive and Brain Sciences in Leipzig. The study was approved by the local Ethics Committee of Leipzig University, Germany (Ethics Approval: Nr 018/23-ek), and conducted in accordance with the principles expressed in the Declaration of Helsinki. All the participants were naïve to the purpose of the experiments and gave written informed consent before the experiment and were compensated for their participation with 13 Euros per hour. All participants had normal or corrected-to-normal vision, with no history of psychiatric or neurological disorders. Due to poor eye-tracking data quality, two participants were excluded from Experiment 1 and one from Experiment 2. In the central-fixation control experiment, one participant failed to complete the task and was excluded. For the remaining 37 participants, fixation compliance was evaluated so that only 31 participants who followed the central-fixation instruction sufficiently well were retained for further analysis.

### Stimuli and tasks

Visual stimuli consisted of either three (Experiment 1 and fixation control experiment) or four (Experiment 2) items presented simultaneously at different locations (6° visual angle eccentricity). Each item (2.8° visual angle in radius) comprised two integrated components: (1) an internal grating with one of six possible spatial frequencies (0.6, 0.9, 1.3, 1.9, 2.7, and 4 cycles per visual degree, logarithmically spaced), and (2) an outer ring in one of six colors (evenly spaced from blue to green in Commission Internationale de l’Eclairage (CIE) LAB color space). A systematic relationship was established between stimulus features: lower spatial frequencies were consistently paired with greener hues, whereas higher spatial frequencies were paired with bluer hues. This created an implicit feature-space structure where color and frequency varied coherently along a single dimension. We used two feature dimensions that varied in a consistent ordinal manner because this creates a richer latent relational structure than either feature alone. Specifically, the shared ordinal relationship is expressed simultaneously across both color and spatial frequency, encouraging participants to represent the stimulus set in terms of its underlying structure rather than as two independent feature dimensions. In this sense, the latent structure is not tied to a particular feature, but to the ordinal relationship shared across features. We reasoned that such a representation would make relational encoding and ordinal sampling more advantageous than memorizing each feature independently, thereby increasing the likelihood that spontaneous structure-based sampling strategies would be expressed in gaze behavior.

#### Experiment 1 (3-item memory).

Each trial began with a central fixation requirement (gaze within 1° for at least 10 ms) to establish a neutral baseline. Three gratings, varying in spatial frequency and color, then appeared simultaneously for 4 s, arranged in an equilateral triangle configuration for encoding. After stimulus offset, participants re-fixate centrally to initiate the 2 s maintenance phase, where three placeholder circles appeared at original stimulus locations. Another central fixation triggered the retro-cue period, where the three circles reappeared with one filled for 100 ms to direct attention to that specific location, followed by another 2 s display of all circles. Note that the cue was intentionally invalid (non-predictive) so that it would not bias participants toward any item in memory, while still exogenously directing attention to a specific location. This allowed us to test more directly whether the subsequent eye movements followed the latent ordinal structure. Participants were explicitly informed before the experiment that the cue was non-predictive. Finally, a test stimulus (grating or color ring) appeared at the center alongside the three circles. Participants were required to indicate which memory item matched the test stimulus by clicking the corresponding location, or to press the “C” key if none of the memory items matched. Therefore, the chance level of memory performance is 0.25. Note that three fixation periods were used to control for spatial biases during memory encoding/maintenance/retro-cue period. Participants needed to first complete a brief practice session of 20 trials to become familiar with the task. Then, the formal experiment comprised 216 trials (6 blocks of ~12 min each) with inter-block eye-tracker calibration, totaling ~100 min (including breaks). As shown in Fig 1B, there were six distinct spatial configurations based on relative stimulus values, with 36 trials for each configuration.

#### Central-fixation control experiment.

This control experiment used the same design as Experiment 1, except that participants were instructed to maintain central fixation throughout the encoding and maintenance periods. To verify compliance, we calculated, for each participant, the proportion of trials in which the maximum gaze deviation from the central fixation point exceeded 2° of visual angle. Six participants showed consistently poor fixation performance (S8A Fig) and were excluded from further analyses. The final sample therefore comprised 31 participants (S8B and S8C Fig).

#### Experiment 2 (4-item memory).

Experiment 2 employed the same stimuli and design as Experiment 1, with one critical modification: four items (instead of three) were simultaneously presented for 5 s, positioned at the vertices of an equilateral rectangle, each possessing distinct spatial frequencies and colors. Following stimulus offset, participants maintained the four items in memory for 2.5 s. To account for the greater cognitive load of the 4-item task compared to the 3-item version, we extended both the encoding duration (from 4 to 5 s) and maintenance period (from 2 to 2.5 s) to ensure adequate information processing. During the response phase, participants indicated either the location matching the test stimulus or pressed “C” for novel items, resulting in a chance performance level of 0.2. Participants also needed to complete 216 trials (6 blocks of ~13 min each) with inter-block eye-tracker calibration. In total, it took around 110 min (including breaks). From the 24 possible spatial configurations in Experiment 2, we selected 12 for testing, with 18 trials per configuration. This necessarily reduced the number of repetitions per configuration compared to Experiment 1 (18 versus 36), which may have lowered sensitivity for configuration-based and sequenceness measures. At the same time, this reduction was a consequence of the task design rather than an arbitrary choice. With four items distributed across four locations, the number of possible arrangements increases substantially. To keep the experiment at a manageable duration while still ensuring that each item had an equal probability of appearing at each location, we selected 12 well-balanced configurations and matched the total number of trials across Experiments 1 and 2 to preserve comparability at the task level. As a result, the number of repetitions per configuration was necessarily lower in Experiment 2 (18 versus 36), which could potentially reduce sensitivity for detecting configuration-specific and sequential gaze structure. To assess the influence of this difference, we additionally conducted a trial-matched analysis in Experiment 1 by randomly subsampling 18 trials from each configuration and repeating the pattern-similarity and sequenceness analyses. The overall pattern of results was consistent with that obtained using all 36 trials per configuration (see S9 Fig).

### Eye-tracking recordings

Participants sat 60 cm from a monitor (1,280 × 1,024 pixels, 60 Hz refresh rate) with their head stabilized using a chin rest. An eye tracker (EyeLink 1000 Plus, SR Research) was positioned on the table approximately 15 cm in front of the monitor. During the task, gaze was continuously tracked for both eyes simultaneously, at a sampling rate of 1,000 Hz. Before acquisition, we calibrated the eye tracker using the built-in calibration and validation protocols from the EyeLink software.

### Eye-tracking preprocessing

Raw eye-tracking data were converted from EDF to MAT format using the Edf2Mat toolbox with default settings. For each trial, we extracted three epochs of interest: (1) the stimulus presentation epoch (−10 to 4,000 ms from stimulus onset), (2) the memory maintenance epoch (−10 to 2,000 ms from placeholder onset), and (3) the memory retrieval epoch (−10 to 2,100 ms from retro-cue onset). Baseline correction was applied using the pre-event interval (−10 to 0 ms). Note that the implementation of baseline correction was to reduce systematic trial-to-trial offsets in gaze position, because the gaze-distance analysis depends on accurately quantifying the spatial relationship between gaze position and the item locations. For each critical period of interest (encoding, maintenance, and retro-cue), we used the immediately preceding fixation interval as the baseline. During these intervals, participants were instructed to fixate at the center of the screen within 1 degree of visual angle from the center for at least 10 ms. Blinks were detected by identifying NaN clusters (≥9 consecutive missing samples) in each epoch. To capture pre/post-blink distortions, we extended each blink window by 100 ms on both sides. Gaze data from both eyes were then averaged, producing horizontal (*x*) and vertical (*y*) gaze time series per epoch. For data quality control, epochs with more than 50% invalid samples were discarded. Remaining blink artifacts were interpolated using MATLAB’s makima method (a smooth cubic Hermite interpolation). Finally, trials where gaze deviated >10° visual angle from central fixation were excluded to ensure proper gaze. Following preprocessing, the mean number of valid trials retained in Experiment 1 was 198 during encoding (SD = 23, range = 108–216) and 196 during maintenance (SD = 25, range = 102–216). In Experiment 2, the mean number of valid trials was 186 during encoding (SD = 26, range = 108–214) and 184 during maintenance (SD = 25, range = 72–216).

### Eye-tracking analysis

#### Basic eye-movement behavior measurement.

Saccades were detected using a velocity-based algorithm [58,59], in which gaze-position data were first transformed into a velocity time series by calculating the Euclidean distance between consecutive samples. Saccade onset and offset were identified when gaze velocity exceeded a threshold of 30° of visual angle per second, with the additional constraints that saccade duration had to fall between 10 and 300 ms and that inter-saccadic intervals were at least 20 ms to ensure distinct, physiologically plausible events. Fixations were defined as events not classified as saccades or blinks and lasting at least 200 ms. Saccade count, fixation count, saccade amplitude, and fixation duration were then separately measured for each task (see S7 Fig).

#### Correlation based on the spatial configuration.

To derive item-specific gaze time courses, we first calculated the Euclidean distance $d_{i}(t)$ between the gaze position and the center of each item i at every time point t. We then converted these absolute distances into a relative gaze-bias measure by mean-centering the distances across items, dividing by the mean gaze-to-item distance, and reversing the sign: $G_{i}(t)\;=-\frac{d_{i}(t)\;-\overset{―}{d}(t)}{\overset{―}{d}(t)}$, where $\overset{―}{d}(t)$, denotes the mean Euclidean distance from gaze to all items at time t. This transformation expresses gaze proximity to each item relative to the other items within the same display. Positive values indicate that gaze was closer to a given item than the average distance to all items, whereas negative values indicate that gaze was relatively farther from that item. Larger values therefore indicate a stronger relative gaze bias toward that item. This procedure produced three item-specific gaze-bias time courses per epoch in Experiment 1 and the central-fixation control experiment (and four in Experiment 2). Note that by quantifying gaze continuously over time, the distance-based analysis can provide complementary information that is not fully captured by event-based measures alone (such as saccades and fixations). We hypothesized that eye-movement trajectories would reflect the spatial configuration of the items, determined by the arrangement of their relative ordinal ranks (e.g., lowest, intermediate, and highest) across the three fixed stimulus locations (as illustrated in Fig 1B, this resulted in six possible spatial configurations). For example, if a low-frequency item appeared at the upper left, a medium-frequency item at the upper right, and a high-frequency item at the bottom, gaze patterns should be more similar to those from trials sharing the same spatial configuration than to those from trials with a different configuration (e.g., high at the upper left, medium at the upper right, and low at the bottom). To test this, we computed pairwise correlations of the normalized gaze-to-item distance time courses across all trial pairs. Correlations were then grouped by whether the trial pair shared the same spatial configuration or not. Higher correlations within same-configuration pairs compared to between-configuration pairs would indicate that gaze trajectories are structured based on the ordinal relationship among stimuli. Note that we averaged the configuration-based correlation values across all items to obtain a single pattern-similarity score per trial pair. This analysis was performed separately for the encoding and maintenance periods. To assess behavioral relevance, we then tested whether individual differences in this pattern-similarity score (i.e., the difference between same- and different-configuration correlations) predicted memory accuracy. The same analysis was repeated in the fixation control experiment and in Experiment 2, with the only difference being that it was applied to four items with 12 different configurations.

#### Sequenceness analysis on memory encoding and maintenance period.

To test whether gaze systematically transitioned between item locations according to their ordinal structure, we applied a sequenceness analysis adapted from the temporally delayed linear modelling (TDLM) framework [19,21] to the normalized item-specific gaze-bias time courses described above. Items were labeled according to their ordinal rank within each trial (Experiment 1: item 1, lowest spatial frequency/greenest hue; item 2, intermediate spatial frequency/hue; item 3, highest spatial frequency/least green hue; the same relative ordering was extended to four items in Experiment 2).

For each participant and task period (encoding and maintenance), we computed the cross-correlation between every pair of item-specific gaze-bias time courses. Specifically, for each pair of items i and j, the gaze-bias time course of item i was correlated with the gaze-bias time course of item j shifted by a series of temporal lags (Δt). This yielded a cross-correlation value for each item pair at each temporal lag. The pairwise cross-correlations were then arranged into an N×N empirical correlation matrix for each lag, where N=3 in Experiment 1 and N=4 in Experiment 2. Each element of this matrix therefore represents the correlation between one item’s gaze-bias time course and another item’s temporally lagged gaze-bias time course. We evaluated temporal lags from 0 to 2,000 ms in Experiment 1 and from 0 to 2,500 ms in Experiment 2.

We next tested whether the pattern of pairwise lagged correlations at each temporal lag corresponded to a hypothesized ordinal sequence. To do so, we compared each empirical correlation matrix with theoretically defined transition matrices representing the forward and reverse ordinal sequences. In Experiment 1, the forward transition model represented the rank-increasing sequence 1 → 2 → 3 and contained transitions from item 1 to item 2 and from item 2 to item 3, whereas the reverse model represented the rank-decreasing sequence 3 → 2 → 1 and contained transitions from item 3 to item 2 and from item 2 to item 1. In Experiment 2, the corresponding forward model represented 1 → 2 → 3 → 4, whereas the reverse model represented 4 → 3 → 2 → 1. At each temporal lag, we quantified the correlation between the empirical lagged-correlation matrix and each theoretical transition matrix. This yielded a sequenceness value as a function of temporal lag for each model. Larger positive values indicate stronger expression of the hypothesized transition structure at that lag. All analyses were performed separately for the encoding and maintenance periods.

To assess statistical significance, we conducted a permutation test by randomly shuffling the item labels within each trial and repeating the sequence analysis on these surrogate datasets. This procedure was repeated 200 times, yielding a null distribution of sequence strength values across temporal lags. A significance threshold (*p* < 0.05, one-sided) was then determined at each lag based on this null distribution. The reason for using a one-sided test is that our sequenceness metric was defined as the positive correspondence between the empirical transition matrix and a given model transition matrix. In other words, the test asked whether the observed transition structure was expressed more strongly than expected by chance in the predicted direction. Importantly, reverse-order effects were not evaluated by negative values of the forward-sequence curve. Instead, they were tested directly using a separate reverse transition model. Thus, positive values for the forward model indicate evidence for the forward sequence, whereas negative values should not be interpreted as evidence for the reverse sequence. To correct for multiple comparisons across lags, we used the maximum value approach by setting the highest threshold across all lags as the corrected significance cutoff.

#### Sequential saccades after a retro-cue.

To examine whether the retro-cue directed gaze toward the cued location, we analyzed the landing position of the first saccade after cue onset, specifically testing whether it was closer to the cued position than to the uncued locations. In trials where the first saccade was directed to the cued location, we further analyzed the subsequent (second) saccade to assess whether it followed an optimal mental scanning trajectory based on item rank order (i.e., item 1 → item 2 → item 3). For example, if the first saccade landed on item 1 (lowest frequency/greenest), the second saccade was expected to target item 2 (middle frequency), rather than item 3 (highest frequency). Similarly, if the first saccade landed on item 2, the second saccade was more likely to shift toward item 3 than item 1, consistent with an ordinal sampling sequence. To test this, trials were selected based on the following criteria: (1) the first saccade was directed to the cued location, (2) the cued item was either item 1 or item 2, and (3) at least two saccades occurred within the trial. Participants with fewer than 20 valid trials based on these criteria (*N* = 4) were excluded from the analysis. For the remaining trials, we compared the forward transitions (item 1 → item 2; item 2 → item3) with alternative transitions (item 1 → item 3; item 2 → item 1), to determine whether the second saccade reflected a structured, optimal sampling strategy. The rationale for focusing initially on trials in which either item 1 or item 2 was cued was that ordered stepwise transitions are most directly interpretable from these starting positions, because they allow a straightforward comparison between the predicted forward transition and alternative transitions. In addition, we conducted a supplementary transition analysis including all cue positions. Across all trials, there were six possible transitions (1 → 2; 1 → 3; 2 → 1; 2 → 3; 3- → 1; 3 → 2), which we grouped into three transition types: forward transitions (1 → 2; 2 → 3), backward transitions (2 → 1; 3 → 2), and long-distance transitions (1 → 3; 3 → 1) (see S5B Fig). A similar analysis was also applied to Experiment 2 (S6C Fig).

## Supporting information

S1 Fig Trail-matched pattern similarity and reverse ordianl sequenceness in Experiment 1. **(A)** To account for unequal trial numbers between the within-pattern and between-pattern conditions in Experiment 1, we randomly subsampled the between-pattern trial pairs to match the number of within-pattern pairs. This procedure was repeated 100 times, and the resulting correlations were averaged across iterations. The pattern similarity effect (mean ± SEM) was then computed separately for the encoding (yellow) and maintenance (purple) periods. *: *p* < 0.05. **(B)** To assess the behavioral relevance of the 3 → 2 → 1 sequence, we compared sequence strength (mean ± SEM) between high (dark red) and low-performance (light red) groups, defined by a median split of memory accuracy (*N* = 23 per group). Results are shown separately for encoding (left panel) and maintenance (right panel). The corresponding data are publicly available on OSF: https://osf.io/kasr2/.
(TIF)

S2 Fig Six possible sequences based solely on spatial locations. To rule out the possibility that the observed sequential eye-movement patterns were driven by systematic transitions between spatial locations rather than the latent ordinal structure, we relabeled items according to their spatial locations and tested all six possible spatial sequences during encoding (**A–C**) and maintenance (**D–F**), using the same sequenceness analysis. No significant sequential patterns were observed, indicating that the original sequential effects were not explained by spatial location alone. The corresponding data are publicly available on OSF: https://osf.io/kasr2/.
(TIF)

S3 Fig Sequence strength of the reverse 4 → 3 → 2 → 1 ordinal sequence in Experiment 2. The reverse ordinal sequence (4 → 3 → 2 → 1) was not significantly expressed during either encoding (**A–Left**) or maintenance (**B–Left**). To examine potential individual differences, participants were divided into high (dark red) and low-performance (light red) groups based on a median split of memory accuracy (*N* = 20 per group). No significant 4 → 3 → 2 → 1 sequence was observed in either group during encoding (**A–Right**) or maintenance (**B–Right**), providing no evidence for reliable expression of the reverse ordinal sequence at either the group or subgroup level. The corresponding data are publicly available on OSF: https://osf.io/kasr2/.
(TIF)

S4 Fig Learning effects across early and late trial halves. To test the learning effect, trials were split into early and late halves. Memory accuracy (**A, G**), pattern-similarity effect (**B, H**), sequential organization (**C–F and I–L**) were separately tested for the early and late halves for Experiment 1(3-item task) and Experiment 2 (4-item task). Error bars indicate SEM. Dots represent individual participants. *: *p* < 0.05. Memory performance (**A**, 3-item task; **G**, 4-item task). Accuracy was significantly higher in the late than in the early half in both tasks (3-item: *t*_(45)_ = 4.57, *p* < 0.001, Cohen’s *d* = 0.67; 4-item: *t*_(39)_ = 5.03, *p* < 0.001, Cohen’s *d* = 0.80). The pattern-similarity effect (**B**, 3-item task; **H**, 4-item task). In the 3-item task, the pattern-similarity effect was present during encoding in both the early and late halves (early: *t*_(45)_ = 5.43, *p* < 0.001, Cohen’s *d* = 0.80; late: *t*_(45)_ = 7.40, *p* < 0.001, Cohen’s *d* = 1.09). During maintenance, the effect was absent in the early half but emerged in the late half (early: *t*_(45)_ = 1.34, *p* = 0.19, Cohen’s *d* = 0.20; late: *t*_(45)_ = 2.90, *p* = 0.006, Cohen’s *d* = 0.43). In the 4-item task, the effect was observed in both halves during both encoding (early: *t*_(39)_ = 8.43, *p* < 0.001, Cohen’s *d* = 1.33; late: *t*_(39)_ = 8.50, *p* < 0.001, Cohen’s *d* = 1.34) and maintenance (early: *t*_(39)_ = 3.03, *p* = 0.004, Cohen’s *d* = 0.48; late: *t*_(39)_ = 2.97, *p* = 0.005, Cohen’s *d* = 0.47). A significant difference between the early and late halves was observed only during encoding in the 3-item task (*t*_(45)_ = 5.23, *p* < 0.001, Cohen’s *d* = 0.77), but not during maintenance in the 3-item task (*t*_(45)_ = 1.33, *p* = 0.19, Cohen’s *d* = 0.20), or during encoding or maintenance in the 4-item task (encoding: *t*_(39)_ = 1.15, *p* = 0.26, Cohen’s *d* = 0.18; maintenance period: *t*_(39)_ = 0.77, *p* = 0.45, Cohen’s *d* = 0.12). Sequential organization (**C–F**, 3-item task; **I–L**, 4-item task). At a descriptive level, the 1 → 2 → 3 sequence during encoding in the 3-item task was present as a trend in the early half, particularly in high-performing participants (**C**), and became more pronounced in the late half (**D**). Similarly, in the 4-item task, the 1 → 2 → 3 → 4 sequential revisitation during maintenance was more clearly expressed in the late half than in the early half, again particularly in high-performing participants (**K, L**). The corresponding data are publicly available on OSF: https://osf.io/kasr2/.
(TIF)

S5 Fig Initial fixation bias during encoding and sequential saccade traansitions following tge retro-cue in Experiment 1. **(A)** To test whether the forward bias might reflect anchoring on the low-frequency item (item 1), we examined the distribution of the first fixation during encoding. Specifically, we quantified how often the first fixation fell closest to item 1 (yellow), item 2 (purple), or item 3 (green). The first fixations were directed significantly more often toward item 1 than expected by chance (chance = 72, *t*_(45)_ = 2.06, *p* = 0.05, Cohen’s *d* = 0.30), whereas fixations toward item 2 (*t*_(45)_ = –5.04, *p* < 0.001, Cohen’s *d* = –0.74) and item 3 (*t*_(45)_ = –1.51, *p* = 0.14, Cohen’s *d* = –0.22) were less frequent. Direct comparisons further showed that first fixations toward item 1 were significantly more frequent than those toward item 2 (*t*_(45)_ = 3.45, *p* = 0.001, Cohen’s *d* = 0.51) and marginally more frequent than those toward item 3 (*t*_(45)_ = 1.86, *p* = 0.07, Cohen’s *d* = 0.28). **(B)** Following the retro-cue, six transitions were possible(1 → 2; 1 → 3; 2 → 1; 2 → 3; 3 → 1; 3 → 2). Transition frequencies were grouped into three types: forward transitions (1 → 2; 2 → 3, yellow), backward transitions (2 → 1; 3 → 2, purple), and long-distance transitions (1 → 3; 3 → 1, green). A significant main effect of transition type was observed (*F*_(2,82)_ = 5.91, *p* = 0.004, *η*_*p*_^2^ = 0.13). Post hoc comparisons showed that forward transitions occurred significantly more often than backward transitions (*t*_(41)_ = 2.36, *p* = 0.04, Cohen’s *d* = 0.32) and long-distance transition (*t*_(41)_ = 3.35, *p* = 0.004, Cohen’s *d* = 0.46), whereas backward and long-distance transitions did not differ significantly (*t*_(41)_ = 0.99, *p* = 0.32, Cohen’s *d* = 0.14). Error bars indicate SEM. Dots represent individual participants. *: *p* < 0.05. The corresponding data are publicly available on OSF: https://osf.io/kasr2/.
(TIF)

S6 Fig Retro-cue related saccade behavior and sequential transitions in Experiment 2. **(A)** First-saccade destination heatmaps for each cue location. Red dashed circles mark the cued locations. **(B)** Number of trials containing two distinct saccades in 3-item (gray) and 4-item (black) tasks. As in Experiment 1, participants with fewer than 20 trials containing two distinct saccades were excluded from this analysis (four participants in Experiment 2). The number of trials containing two distinct saccades was significantly lower in Experiment 2 than in Experiment 1 (Fig B; *t*_(76)_ = −3.54, *p* = 0.001, Cohen’s *d* = −0.80). **(C)** Number of trials containing forward (1 → 2; 2 → 3; 3 → 4) and backward transitions (2 → 1; 3 → 2; 4 → 3) in Experiment 2. Forward transitions did not occur significantly more frequently than backward transitions (Fig C, *t*_(35)_ = −0.03, *p* = 0.98, Cohen’s *d* = −0.005), nor did they occur more often than expected by chance (*t*_(35)_ = 0.79, *p* = 0.44, Cohen’s *d* = 0.13). Error bars indicate SEM. Dots represent individual participants. *: *p* < 0.05. The corresponding data are publicly available on OSF: https://osf.io/kasr2/.
(TIF)

S7 Fig Basic eye-movement measures and fixation-sequence analysis in the 3-item and 4-item tasks. **(A)** Number of saccades during encoding in the 3-item (yellow) and 4-item (purple) tasks. Participants made more saccades in the 4-item than in the 3-item task (*t*_(84)_ = 6.94, *p* < 0.001, Cohen’s *d* = 1.50). **(B)** Number of fixations during encoding. More fixations were made in the 4-item compared to 3-item task (*t*_(84)_ = 7.19, *p* < 0.001, Cohen’s *d* = 1.55). **(C)** Saccade amplitude during encoding. A marginally significant difference was observed between the two tasks (*t*_(84)_ = 1.92, *p* = 0.06, Cohen’s *d* = 0.42). **(D)** Fixation duration during encoding. The two task showed no significant difference (*t*_(84)_ = 0.91, *p* = 0.36, Cohen’s *d* = 0.20). **(E–H)** similar to (ABCD), but for the maintenance period. The 4-item task showed a greater number of saccades (E; *t*_(84)_ = 2.58, *p* = 0.01, Cohen’s *d* = 0.56) and fixations (F; *t*_(84)_ = 3.34, *p* = 0.001, Cohen’s *d* = 0.72) than 3-item task. No significant differences were observed in saccade amplitude (G; *t*_(84)_ = 1.42, *p* = 0.16, Cohen’s *d* = 0.31) or fixation duration (H; *t*_(84)_ = 0.27, *p* = 0.79, Cohen’s *d* = 0.06). **(I)** Fixation-sequence analysis in the 3-item task. Left: fixation order showed evidence of a 1 → 2 → 3 sequence relative to chance level (36) during encoding (yellow, *t*_(45)_ = 2.76, *p* = 0.008, Cohen’s *d* = 0.41), but not during maintenance (purple). Right: during encoding, this sequential fixation pattern was present in high-performing participants (*t*_(22)_ = 3.40, *p* = 0.003, Cohen’s *d* = 0.71), but not in low-performing participants (*t*_(22)_ = 0.28, *p* = 0.78, Cohen’s *d* = 0.06). **(J)** Same as (I), but for 4-item task. The 1 → 2 → 3 → 4 sequence pattern wasn’t significantly expressed at group level (chance level: 18, *t*_(39)_ = 1.51, *p* = 0.14, Cohen’s *d* = 0.24), but significantly expressed in high-performing participants (high-performing: *t*_(19)_ = 2.31, *p* = 0.03, Cohen’s *d* = 0.52; low-performing: *t*_(19)_ = –1.39, *p* = 0.18, Cohen’s *d* = –0.31). Error bars indicate SEM. Dots represent individual participants. * *p* < 0.05. Note that no reliable fixation sequence was detected during maintenance, likely because many trials contained few overt saccades and fixations. The corresponding data are publicly available on OSF: https://osf.io/kasr2/.
(TIF)

S8 Fig Gaze position during encoding and maintenance in the central-fixation control experiment. **(A)** Gaze position time courses along the horizontal (upper panel) and vertical (lower panel) axes during the encoding for the six participants who were excluded because they did not sufficiently maintain central fixation. **(B)** Gaze position time courses along the horizontal (upper panel) and vertical (lower panel) axes during the encoding period for the remaining 31 participants included in the analyses. **(C)** Heat maps of the overall gaze distribution for the 31 included participants during encoding (left) and maintenance (right), demonstrating successful fixation at the center of the screen. The corresponding data are publicly available on OSF: https://osf.io/kasr2/.
(TIF)

S9 Fig Subsampling Experiment 1 to match the number of trials per configuration in Experiment 2. To assess whether the larger number of trials per configuration in Experiment 1 contributed to the observed effects, we randomly subsampled 18 of the 36 trials from each spatial configuration, matching the number of trials per configuration in Experiment 2, and repeated the pattern-similarity and sequenceness analyses. **(A)** Pattern-similarity effect during encoding (*t*_(45)_ = 7.77, *p* < 0.001, Cohen’s *d* = 1.62) and maintenance (*t*_(45)_ = 2.11, *p* = 0.04, Cohen’s *d* = 0.44). **(B)** Sequenceness during encoding. Left: sequence strength for the 1 → 2 → 3 and 3 → 2 → 1 sequences across all participants. Right: 1 → 2 → 3 sequence strength shown separately for high- and low-performing participants, defined by a median split of memory performance. **(C)** Same as (B), but for the maintenance period. Overall, the trial-matched dataset showed a pattern consistent with that observed in the full Experiment 1 dataset. The corresponding data are publicly available on OSF: https://osf.io/kasr2/.
(TIF)

## Abbreviation

TDLM: temporally delayed linear modelling
