# Supplementary material for: Eye movements reflect capacity-dependent organization of working memory
Source: PLoS Biol. 2026 Oct 13;24(10):e3004047. doi: 10.1371/journal.pbio.3004047 (PMC13674860; doi:10.1371/journal.pbio.3004047)
Supplement: Supplementary file 1 [file pbio.3004047.s010.docx]

**Reviewer remarks:**

Reviewer #1 Andrey R. Nikolaev (note, reviewer 1 has signed this review): This manuscript tests whether eye movements spontaneously impose an ordinal structure on working-memory representations under different task demands. Participants performed a working-memory task in which each item combined a colored ring and a grating, with a consistent color-spatial-frequency pairing that defined a latent ordinal structure. In Experiment 1 (3 items, low demand) and Experiment 2 (4 items, high demand), 89 participants encoded and maintained the ordinal structures, while their eye movements were recorded. At the end of the trial, their item memory was tested. The authors analyzed configuration-specific similarity of gaze-item distance trajectories and direction-sensitive "sequenceness" metrics during encoding and maintenance. They then related these measures to subgroups defined by accuracy and performance. The authors found that eye movement patterns aligned with the latent ordinal structure. The alignment depended on mental load. In the low-load condition, the structure emerged during encoding; in the high-load condition, it was more prominent during maintenance. The authors concluded that eye movements actively organize working memory.

I like this study, especially the elegant way of using a latent, ordered feature structure and eye tracking to examine how memory representations are sampled. The main conclusion that gaze helps organize working memory is important and very timely. However, before the main claims can be fully made, I'd like to ask for a few clarifications. Particularly, the study would benefit from: strengthening the evidence for "demand-dependent memory expression" in the Exp1 vs Exp2 comparison; testing why the value-increasing sequence is stronger than the reverse one; tempering interpretation of highly selected analyses/subgroups in Exp2, and clarifying several points in the method.

We thank the reviewer for the positive feedback and for the thoughtful, constructive comments. We have carefully considered each point and believe that addressing these suggestions has strengthened the manuscript. Our detailed responses are provided below.

In Experiment 1, the value-increasing (forward) sequence was found to be much stronger than the reverse sequence. However, both directions seem equally probable. The Discussion's suggestion that participants "anchor" on low-frequency items (and the speculative link to magnitude/mental number line theories) is plausible, but not supported by tests. Since directional asymmetry is the central empirical feature, it would benefit from direct testing. For example, one could test whether the directionality is explained by anchoring (distribution of sequence start items), spatial layout confounds (weak correlation between items and particular positions), and/or oculomotor costs (saccade amplitude/nearest-neighbor transitions).

We thank the reviewer for this important point. We agree that the directional asymmetry between the forward and reverse sequences is a central feature of the results and benefits from direct testing. To address the possibility that the forward bias reflects anchoring on low-frequency item, we examined the distribution of the first fixation during encoding. Specifically, we tested how often the first fixation fell closest to item 1 (lowest spatial frequency / greenest), item 2 (intermediate spatial frequency / colour), or item 3 (highest spatial frequency / least green). We found that the first fixation was directed significantly more often toward item 1 than expected by chance (t _(45)_ =  2.06, p = 0.046, Cohen’s d = 0.30), whereas fixations toward item 2 (t _(45)_ =  –5.04, p < 0.001, Cohen’s d = –0.74) and item 3 (t _(45)_ =  –1.51, p = 0.14, Cohen’s d = –0.22) were less frequent. Direct comparisons further showed that first fixations toward item 1 were significantly more frequent than those toward item 2 (t _(45)_ =  3.45, p = 0.001, Cohen’s d = 0.51) and marginally more frequent than those toward item 3 (t _(45)_ =  1.86, p = 0.07, Cohen’s d = 0.28). This result is consistent with the idea that the forward sequence may be partly driven by anchoring on the low-frequency item. These findings indicate that participants preferentially initiated sampling from the lowest-frequency item, which is consistent with the proposed anchoring account. We have added this result in supplementary figure 5A.

We also considered whether the directional asymmetry could be explained by spatial layout or by the differences in oculomotor cost. In the present design, the three items were positioned at the vertices of an equilateral triangle, such that the distance between any pair of items was identical. As a result, the total scan-path distance is the same for forward and reverse sequences, making it unlikely that the observed forward bias can be explained by lower oculomotor cost. In addition, the assignment of three items to the three spatial locations was fully counterbalanced, so there was no systematic mapping between ordinal position and physical position. These aspects of the design make a spatial-layout or oculomotor cost explanation unlikely.

At the same time, we agree that the present data do not fully resolve why participants preferred the forward over the reverse direction. In addition to the possible link with magnitude-based or mental-number-line accounts, another possibility, as Reviewer 2 notes, is that the low-frequency item may have greater perceptual salience and therefore served as a natural anchor during encoding. However, we do not think the current findings can be fully explained by saliency alone, for two reasons. First, the structured sequence reappeared during the maintenance period, when no visual input was present, indicating that the effect was at least partly memory-driven rather than purely stimulus-driven. Second, the expression of the sequence changed as a function of task stage and memory demand, which is more consistent with flexible cognitive organization than with a fixed low-level visual bias. Taken together, we think the current findings are more consistent with an active memory-organization account than with a purely saliency-driven explanation, while acknowledging that low-level salience may contribute to the initial encoding bias.

We have therefore revised the Discussion to treat anchoring/salience as a plausible contributing factor (Page 21 & 22).

Furthermore, the core interpretation assumes that participants learned this directionality. But it is unclear how quickly participants learned it during the experiment (without a practice session) and how consistent learning was for high and low performers. Please add a time-course analysis (e.g., early vs late blocks, or trial-wise learning curves) for the key measures (pattern similarity, encoding/maintenance sequenceness, and accuracy).

We thank the reviewer for this helpful suggestion. To clarify, participants completed a brief practice session of 20 trials before the formal experiment to become familiar with the task (added to the Methods, Page 23). To further examine how quickly they picked up on the latent directional structure, we divided the experiment into early and late halves and compared memory performance and gaze-based measures across these two phases.

Memory performance was significantly higher in the late than in the early half for both tasks (3-item: t _(45)_ = 4.57, p < 0.001, Cohen’s d = 0.67, Figure A; 4-item: t _(39)_ = 5.03, p < 0.001, Cohen’s d = 0.80, Figure G), suggesting some improvement over the course of the experiment.

Importantly, however, the pattern-similarity effect was already present in the early half of the experiment (Figure B and H). Specifically, in both the 3-item and 4-item tasks, trials drawn from the same configuration pattern showed greater trajectory similarity than trials drawn from different patterns even in the early half (3-item, encoding period: t _(45)_ =  7.13, p < 0.001, Cohen’s d = 1.05, maintenance period: t _(45)_ =2.73, p = 0.009, Cohen’s d = 0.40; 4-item: encoding period: t _(39)_ = 10.00, p < 0.001, Cohen’s d = 1.58, maintenance period: t _(39)_ = 4.35, p < 0.001, Cohen’s d = 0.69). No reliable differences were observed between the early and late halves for this effect (3-item, encoding period: t (45) =  0.01, p = 0.99, Cohen’s d = 0.002, maintenance period: t _(45)_ =  –0.41, p = 0.68, Cohen’s d = –0.06; 4-item, encoding period: t _(39)_ = –0.20, p = 0.84, Cohen’s d = –0.03), with the exception of a marginal difference in the 4-item maintenance period, where the early half showed a somewhat stronger effect than the late half (t _(39)_ = 1.92, p = 0.06, Cohen’s d = 0.30). This suggests that participants were sensitive to the latent organization relatively early in the task, rather than only acquiring it gradually after extended exposure. This is also consistent with our design rationale, namely to make the latent structure sufficiently obvious that participants could readily detect and exploit it.

Meanwhile, it is important to note that the pattern-similarity analysis reflects sensitivity to the latent ordinal structure but does not by itself require a stable1-2-3 or 1-2-3-4 sequential sampling pattern. The sequenceness analysis addresses this stricter question by testing whether the latent structure is expressed as a stable ordered sampling sequence over time. We therefore also applied the sequenceness analysis separately to the early and late halves of the experiment (Figure CDEF for 3-item task and Figure IJKL for 4-item task). At a descriptive level, the 1-2-3 sequence during encoding in the 3-item task was already visible as a trend in the early half, particularly in high-performing participants, and became more pronounced in the late half. Similarly, in the 4-item task, the 1-2-3-4 sequential revisitation during maintenance appeared more clearly in the late half than in the early half, again particularly in high-performing participants. These comparisons should be interpreted cautiously, however, because splitting the data substantially reduced statistical power.

Taken together, these analyses suggest that participants were already sensitive to the latent structure early in the experiment, while the more stable ordered eye-movement sequence appears to have become more strongly expressed with experience across trials. Thus, the data are consistent with an early acquisition of the latent structure, together with some subsequent refinement of the sequential sampling strategy over time.

We have incorporated these analyses into the Results (Pages 6–7) and reported the corresponding findings in supplementary figure 4.

In relation to this, Figs. 2D,E; 3G,H show a single dashed line indicating a permutation-derived p<0.05 threshold above 0. But the y-axis is a correlation coefficient, whose negative values can be potentially interpretable (a reverse sequence?). The Methods describe that the threshold was obtained in label-shuffling, but do not state whether the permutation test was one- or two-sided. Please specify the sidedness explicitly. If the test was one-sided, please justify the directional hypothesis and clarify how reverse-order effects are evaluated.

We thank the reviewer for pointing this out. The permutation test was one-sided. Specifically, for each hypothesized transition model, we tested whether the sequenceness value in the empirical data was significantly greater than the permutation-derived null distribution by label shuffling. We used a one-sided test because our a priori hypothesis was that participants would exhibit the predicted sequential organization, resulting in greater correspondence with the hypothesized transition model than expected by chance.

Reverse-order effects were not evaluated by negative values of the forward-sequence curve. Instead, they were tested directly using a separate reverse transition model. Thus, positive values for the forward model provide evidence for the forward sequence, whereas evidence for the reverse sequence is assessed independently using the reverse model. Consequently, negative values of the forward-model curve should not be interpreted as evidence for reverse replay.

The negative values observed at very short lags are most likely attributable to strong autocorrelation in the gaze time series. Near zero lag, the empirical transition matrix is dominated by the diagonal elements (r₁₁, r₂₂, r₃₃), which primarily reflect self-similarity rather than genuine sequential transitions. Consistent with this interpretation, these negative values did not exceed the lower bound of the permutation distribution and therefore were not statistically significant (see example below).

We have clarified in the Methods that the permutation test was one-sided and have added an explanation of how reverse-order effects were evaluated using a separate reverse transition model (Page 28).

The retro-cue analysis excludes trials where item 3 is cued ("only item 1 or item 2 … was cued"). The authors explained this by saying that ordered transitions are "interpretable" only from start positions 1 or 2. However, this restriction creates the risk of introducing predicted directionality. If the claim is truly an ordered structure, then transitions from item 3 (3-2 and 3-1) are also diagnostic and should be analyzed. Please justify this exclusion more explicitly and/or include a fuller transition analysis across all cue positions.

We thank the reviewer for this helpful suggestion. We agree that restricting the original analysis to trials in which item 1 or item 2 was cued may give the impression that the analysis was biased toward the predicted forward direction. Our original rationale was that ordered stepwise transitions are most directly interpretable when the starting point is item 1 or item 2, because these positions allow a straightforward comparison between the forward transition and alternative transitions. However, we agree that transitions beginning from item 3 are also informative and should be reported more fully.

We therefore conducted an additional transition analysis including all cue positions. Across all trials, there were six possible transitions (1-2; 1-3; 2-1; 2-3; 3-1; 3-2). We grouped them into three transition types: forward transitions (1-2; 2-3), backward transitions (2-1; 3-2), and long-distance transitions (1-3; 3-1). As shown below, we found significant main effect of transition type (F_(2,82)_ = 5.91, p = 0.004, ηₚ² = 0.13). Post hoc comparisons showed that forward transitions occurred significantly more often than backward transitions (t _(41)_ = 2.36, p = 0.04, Cohen’s d = 0.32) and long-distance transition (t _(41)_ = 3.35, p = 0.004, Cohen’s d = 0.46), whereas backward and long-distance transitions did not differ significantly from each other (t _(41)_ = 0.99, p = 0.32, Cohen’s d = 0.14).

These results indicate that the forward transition is not an artifact of excluding item-3-cued trials, but is also present when all possible starting positions are taken into account. We have added this analysis to Results (Page 10) and supplementary figure 5B and clarified the rationale for the original analysis in Methods (Page 29).

In addition, the retro-cue analysis is absent in Experiment 2, but it would strengthen the "demand-dependent structure" claim.

We thank the reviewer for this suggestion. We have now conducted the retro-cue analysis in the 4-item task, and the results are summarized below.

As in Experiment 1, the majority of first saccades were still directed toward the cued location (Figure A, t _(39)_ = 3.90, p < 0.001, Cohen’s d = 0.61). We then examined the second-saccade transitions. The 4-item task contains a larger set of possible transitions. We classified the second-saccade transitions into forward transitions (1-2; 2-3; 3-4), backward transitions (2-1; 3-2; 4-3), and other/non-adjacent transitions (1-3; 1-4; 2-4; 3-1; 4-1; 4-2). Unlike Experiment 1, forward transitions in Experiment 2 did not occur more frequently than backward transitions (Figure C, t_(35)_ = –0.03, p = 0.98, Cohen’s d = –0.005), nor did they occur more often than expected by chance (t_(35)_ = 0.79, p = 0.44, Cohen’s d = 0.13, chance = 3/12).

This null result should, however, be interpreted with some caution. As in Experiment 1, participants with fewer than 20 trials containing two distinct saccades were excluded from this analysis (four participants in Experiment 2). More importantly, trials containing two distinct saccades occurred significantly less frequently in Experiment 2 than in Experiment 1 (Figure B; t _(76)_ = −3.54, p = 0.001, Cohen's d = −0.80). Because the retro-cue transition analysis specifically relies on multi-step saccade sequences, the reduced number of such trials substantially decreases the sensitivity and statistical power of this analysis. In addition, the 4-item task doubles the number of possible pairwise transitions compared with the 3-item task, further reducing the likelihood of observing consistent transition patterns. Together, these factors likely contributed to our inability to detect a reliable structured second-saccade transition pattern in Experiment 2. Future studies with a larger number of trials will therefore be important for determining whether structured transition patterns can be detected more reliably under higher memory load.

We have added these results in supplementary figure 6 (Page 28).

In Experiment 2, the key sequential signature at encoding (1-2-3-4 sequenceness) is not statistically significant. Specifically, the results indicate that gaze reflects the value-guided structure (configuration-sensitive pattern similarity), but they do not establish encoding sequenceness in the 4-item task (Fig. 3G,H). Yet the Discussion interprets the maintenance sequenceness as a "shift toward compensatory reorganization" or "replay" of the same sequence as at encoding. This is stronger than what the data directly support. I suggest either tempering the 'replay' language, or adding analyses that explicitly link encoding and maintenance sequenceness.

We thank the reviewer for this important point. We agree that the original wording was too strong. In Experiment 2, although the configuration pattern effect indicates that gaze remained sensitive to the latent ordinal structure (Figure 5C&E), the 1-2-3-4 sequenceness effect during encoding was not statistically significant (Figure 5G). We therefore agree that the maintenance-period sequenceness effect should not be described as a direct “replay” of an encoding sequence.

We have revised the manuscript accordingly throughout, including the title, abstract, and main text, and now adopt a more cautious interpretation. Rather than describing the maintenance-period effect in Experiment 2 as a replay of the same sequence observed during encoding, we now interpret it as evidence for post-encoding reorganization or structured revisitation during maintenance. This interpretation is more consistent with the present data, which demonstrate sensitivity to the latent ordinal structure in Experiment 2 but do not provide equally strong evidence for a stable ordered encoding sequence at the group level.

Experiment 2 uses half as many trials per configuration than Experiment 1 (18 vs 36). This likely reduces sensitivity for configuration-based measures and could contribute to weaker effects at encoding in Experiment 2. Please discuss this limitation and/or provide a power/sensitivity check (e.g., Experiment (1 vs 2) x Phase (encoding vs maintenance) interaction on sequenceness) showing that the sequenceness metric is comparably detectable across two experiments.

We thank the reviewer for this helpful point. We agree that Experiment 2 included fewer trials per configuration than Experiment 1 (18 vs. 36), which likely reduced sensitivity for both the configuration-based and sequenceness analyses. This is therefore an important limitation when comparing the strength of the effects across the two experiments. At the same time, we note that reduced trial numbers alone are unlikely to fully account for the observed pattern in Experiment 2, as robust maintenance-related sequential reorganization was still evident in the high-performing participants.

The lower number of repetitions per configuration in Experiment 2 was a consequence of the task design. With four items placed at four locations, the number of possible arrangements increases substantially. To keep the experiment at a manageable duration while ensuring that each item had an equal probability of appearing at each location, we selected 12 well-balanced configurations. At the same time, we matched the total number of trials across Experiments 1 and 2 to preserve overall comparability at the task level. As a result, however, each configuration was presented fewer times in Experiment 2.

We have revised the manuscript to discuss this limitation explicitly (Page 24).

Minor points

The cue was invalid: it is shortly mentioned only in the Fig. 1 caption, but this should be explained (with motivation) in Methods.

We thank the reviewer for pointing this out. The cue was intentionally made invalid (non-predictive) so that it would not bias participants toward any particular item in memory, while still exogenously directing attention to a specific location. This allowed us to test more directly whether the subsequent eye movements followed the latent ordinal structure rather than simply reflecting strategic use of a predictive cue. Participants were explicitly informed before the experiment that the cue was non-predictive. We have now clarified both the rationale and the procedure in the Methods (Page 23).

The paper mentions "re-visitation" in several places, but it is not clear how it was defined and measured and how it relates to the literature on gaze refixations, particularly those related to memory encoding (e.g., Kragel & Voss 2022; Nikolaev et al. 2025).

We thank the reviewer for raising this point. We agree that the term re-visitation should be defined more clearly and more explicitly related to the literature on gaze refixations.

In the present study, we use re-visitation to refer to eye movements that return to the remembered locations of previously presented items during the maintenance period, after the stimuli have disappeared from the screen. This was quantified by computing the gaze-distance time courses relative to the former item locations and examining their temporal order using the same analysis framework as during encoding. Because the items were no longer visible, these eye movements should reflect internally guided sampling.

We agree that this phenomenon is closely related to the broader literature on gaze refixations. Both involve repeated sampling of previously viewed locations and have been proposed to support memory encoding and retrieval (e.g., Kragel & Voss, 2022; Nikolaev et al., 2025). We use the term re-visitation to emphasize that, in our paradigm, these repeated fixations occurred after encoding, during the maintenance period, when gaze was directed to the remembered rather than visible locations. Thus, we view re-visitation as a memory-guided form of refixation rather than as a distinct phenomenon.

We have revised the Discussion (Page 20) to clarify this terminology, explicitly relate our findings to the literature on gaze refixations, and cite the relevant studies.

In Experiment 1 the way of split into high/low performers is explicitly defined. In Experiment 2, the Results refer to "high-performing participants" and Fig. 4 implies a high/low split (N=20 per group), but the grouping procedure is not described in the text.

We apologize that this was not stated clearly in the original text. In Experiment 2, we applied the same median-split procedure as in Experiment 1 to divide participants into high- and low-performance groups.

We have now clarified this in the Results section (Page 14).

Methods: Eye-tracking preprocessing and analysis:

Specify in more detail why baseline correction was needed and how it was done.

We thank the reviewer for this suggestion. Baseline correction was applied to compensate for small trial-to-trial offsets in eye position arising from calibration drift and natural variability in fixation. This step was important because our gaze-distance analysis depends on accurately quantifying the spatial relationship between gaze position and the item locations. For each critical period of interest (encoding, maintenance, and the retro-cue period), we used the immediately preceding fixation interval as the baseline, during which participants were instructed to maintain central fixation. A baseline was accepted only if gaze remained within 1° of visual angle from the center for at least 10 ms. The mean gaze position during the accepted baseline period was then subtracted from the subsequent gaze coordinates on that trial before computing gaze distance.

We have clarified this procedure in the revised Methods (Page 25).

Give also the range of numbers of valid trials retained.

We thank the reviewer for this helpful suggestion. We have now added the range of valid trials retained for each experiment to the Methods (Page 25).

From which part of the item was the Euclidean distance to the gaze position measured?

We apologize that this was not stated clearly in the original manuscript. The Euclidean distance was computed from the center of each item to the gaze position. We have now clarified this in the Methods section (Page 26).

Reviewer #2: The investigators used two working memory tasks in investigate the effects of eye movements on memory performance. Their stimuli had two features (spatial frequency grating and colour) that were matched in an ordinal way such that they progressed from greenest and lowest spatial frequency to the bluest and highest spatial frequency.

They first showed that eye movements were more similar within a particular presentation pattern than between patterns. They then used a state space analysis to show that in general there is a strategy to first sample the lowest frequency/greenest target and then proceed along the ordinal hierarchy. This effect was stronger in higher performing participants during encoding, while they did not use the sequence during maintenance. Lower performing participants had similar sequence correlation in encoding and retrieval. The experimenters also showed that when an uninformative cue was used to attract attention, the subsequent saccade was toward the next object in the sequence.

They also used a 4-item task with the same structure, replicating the pattern analysis but showing that it was weaker at encoding and stronger during maintenance, relating this to task demands increasing which disrupts efficient encoding, and relies on reorganizational processes during maintenance. They also redid the state space analysis and again showed that the sequence was weak during encoding, and stronger during maintenance.

They have used some interesting methods to investigate eye movements during a working memory task, but I have some reservations.

We thank the reviewer for the careful assessment of our manuscript and for the insightful, constructive comments. We have carefully considered each suggestion and revised the manuscript accordingly. Our point-by-point responses are provided below.

Major concerns

They find a strategy to sample in a sequence from low to high spatial frequency, which is called 'value', but is likely following a salience gradient (Urban et al., 2011). The fact they did not find the reverse pattern in some people suggests salience is driving the pattern.

We thank the reviewer for raising this concern. We agree that low-level visual salience is an important alternative explanation. While Urban et al. (2011) showed that spatial-frequency content can influence fixation allocation, with relatively low spatial frequencies often exerting a stronger influence on gaze attraction (e.g.,0.7 to 1.3 cycles per degree for nature scenes, 1.3 to 2.7 cycles per degree for man-made street scenes), their study focused on where observers fixated during free viewing rather than how gaze was sequentially organized across fixations. Thus, their findings could plausibly explain an initial orienting bias toward a lower-frequency item, but they do not by themselves explain the ordered low-to-high sampling sequence observed in our study.

At the same time, the comparison with Urban et al. should be made cautiously. Their study examined gaze allocation in complex natural scenes, where spatial frequency was embedded within objects, textures, edges, and scene layout, whereas our stimuli consisted of simple gratings in which spatial frequency was manipulated directly and presented in a tightly controlled display. Moreover, the three items in our task were randomly selected from six possible spatial frequencies (0.6, 0.9, 1.3, 1.9, 2.7, and 4 cycles/degree), and the ordinal labels were assigned according to their relative frequencies within each trial. Consequently, the same spatial frequency could serve as the lowest-frequency item on one trial but as the highest-frequency item on another. It is therefore not straightforward to relate our stimuli to the findings of Urban et al., as it remains unclear whether any salience effect should be determined by the absolute spatial frequency of a stimulus or by its relative frequency within the current display.

The reviewer also notes that the absence of a reverse sequence may be consistent with a salience-based account. We agree that this observation alone does not distinguish between a salience-driven and a memory-guided explanation. Rather, our interpretation is based on both encoding and maintenance findings rather than on the presence or absence of a reverse sequence alone. Specifically, two observations argue against a purely bottom-up salience account. First and foremost, the structured sampling sequence re-emerged during the maintenance period, when no visual input was present, indicating that the effect was at least partly memory-driven rather than purely stimulus-driven. Second, the expression of the sequence changed systematically across task stages and as a function of memory load, which is more consistent with flexible cognitive organization than with a fixed salience gradient. **Taken together, these findings suggest that low-level visual salience may bias the initial orienting of gaze but is unlikely to account for the full sequential organization observed in our experiments.**

We have therefore revised the Discussion (Page 21) to explicitly acknowledge low-level visual salience as a plausible contributor to the initial orienting of gaze while clarifying why we do not consider it a sufficient explanation for the sequential organization observed in our experiments.

In a three-item task, item two is always adjacent to item one, which is not the case in a four-item task. Going to the most salient item adjacent to the first item is a probable strategy that would not follow the 'value' sequence when sampling time is limited.

We thank the reviewer for this additional point. We agree that the spatial arrangement of the items differs between the two tasks. In the 3-item task, the three items were positioned at the vertices of an equilateral triangle, so that all pairwise distances were identical. In contrast, the 4-item task included both neighboring and diagonal transitions, raising the possibility that observers might preferentially make shorter saccades when sampling time is limited.

We do not exclude the possibility that transition distance may influence eye-movement strategy during encoding. However, we think it is unlikely to account for the weaker ordinal sequence observed in the 4-item task. First, the spatial configurations were fully counterbalanced, such that each ordinal item was equally likely to appear at each location and to be adjacent to every other ordinal position across trials. More importantly, the sequential reorganization pattern observed during the maintenance period, when no visual stimuli were present, suggests that the sequence is guided by an internally generated organization based on ordinal structure rather than by the spatial properties of the visual display.

We therefore believe that the weaker sequential effect observed in the 4-item task is more likely to reflect the increased cognitive demands of encoding four rather than three items, together with the larger number of possible transition paths. Both factors are expected to reduce the consistency with which a structured multi-step sampling strategy is expressed overtly within the available time.

The state space analysis appears to be overly complex. It delivers a particular sequence and transition time, but the time is not addressed, and it does not appear to address the variance between participants in sampling time. The authors demonstrate that they are capable of doing an item by item fixation analysis in the non-informative cue first and second saccade analysis. This is sufficient for their claims.

We thank the reviewer for this comment. The primary purpose of our sequenceness (state-space) analysis was to quantify whether eye movements exhibited a structured ordinal organization over time and how this organization changed as a function of task demands. It was not intended to provide precise estimates of the transition time considering the variability in sampling dynamics across participants. Accordingly, we did not make strong claims about the absolute timing of transitions in our manuscript.

We agree that estimating participant- or trial-specific transition dynamics would be an interesting direction for future work. However, reliably characterizing such fine-grained temporal differences would likely require substantially more trials and a higher signal-to-noise ratio than were available in the present study.

We also agree that the first- and second-saccade analyses following the non-informative retro-cue provide a direct and intuitive item-by-item demonstration of the same underlying organizational principle. However, these analyses capture only the saccades following an externally presented cue and therefore cannot characterize the continuous evolution of sampling throughout the encoding period or the spontaneous sequential re-visitation observed during maintenance. In contrast, the sequenceness analysis makes use of the entire gaze time course and provides a unified framework for quantifying sequential organization across both encoding and maintenance. We therefore view the two approaches as complementary, with the item-by-item saccade analysis providing an intuitive validation of the sequential organization revealed by the sequenceness analysis, rather than replacing it.

The description of the 4-item task being more demanding is questionable. Yes, they demonstrate that performance is decreased, but chance is also decreased. From the figure, performance appears to drop from ~66 to ~58, as a ratio of chance, this is actually better performance for 4 items (5 responses) at 2.9x chance vs 2.64x chance for the 3 item (4 responses). There are no numbers reported, so I cannot confirm the method used, but it appears it might just be performance - chance, which does not appear fair to me. 70% performance on a 50% chance task should not be equivalent to 30% performance on a 10% chance task. Both are 20% above chance but I would expect a classifier capable of the latter performance to be above 70% on the first task. This is critical because the entire claim of difference in task demands relies on this.

We thank the reviewer for raising this important concern. We agree that raw accuracy is not directly comparable between the 3-item and 4-item tasks because the corresponding chance levels differ (25% vs. 20%). We also agree that simply subtracting chance is not an appropriate normalization, as equal absolute deviations above chance do not correspond to equivalent levels of performance when the number of response alternatives differs.

To address this concern, we analyzed performance using chance-corrected accuracy, (p-c)/(1-c), where p is observed accuracy and c is the chance level. This measure rescales performance relative to the interval between chance and perfect performance and and can be interpreted as the proportion of trials on which participants responded correctly beyond what would be expected from guessing. Using this measure, performance in the 4-item task remained lower than in the 3-item task, although the difference was modest, (t(84)=1.62, p = 0.05, one-tailed, Cohen’s d = 0.35).

We have revised the manuscript accordingly and now present this comparison more cautiously. Specifically, we no longer interpret the raw accuracy difference alone as evidence that the 4-item task was more demanding. Rather, our conclusion is based primarily on the experimental manipulation itself: by design, participants were required to maintain one additional item in the 4-item task, increasing the nominal working-memory load. The chance-corrected behavioral comparison provides convergent, albeit modest, support for this interpretation.

We have added the corresponding statement to the Results section (Page 13).

The claim that eye movements actively organize memory appears to stem from differences in strategy of those who perform better or worse. I do not think this is sufficient. Eye movements are not controlled for. They could either be not allowed, or instructed, either of which, with the right results, could indicate that the eye movements organize anything. It is perfectly reasonable that a covert attentional spotlight could operate in the same sequence manner, but in the absence of overt saccades, and still have similar performance. In this case, it would show that sequential schemas are supporting memory. In this experiment, EEG/MEG could be used with the TDLM method to explore when participants were paying attention to specific locations. While I think that there is interesting results to be extracted from the current data, I think that claims that saccades organize memory is not supported.

We thank the reviewer for raising this important concern. We agree that the relationship between spontaneous eye-movement patterns and memory performance, by itself, does not establish that eye movements causally organize memory. As the reviewer notes, such patterns could reflect broader strategic differences between participants, or could be an overt manifestation of an underlying covert attentional sequence.

To address this issue more directly, we conducted a new control experiment in which participants performed the same 3-item task while fixating at the center of the screen throughout the encoding and maintenance periods (shown below).

Thirty-seven participants completed this control experiment. After excluding participants who did not maintain central fixation sufficiently, the final sample comprised 31 participants (see supplementary figure 8). Compared with the free-viewing condition (Experiment 1), memory performance was significantly worse under this fixation constraint (Figure A, t _(75)_ = 4.37, p < 0.001, Cohen’s d = 1.01). In addition, the configuration-specific pattern-similarity effect observed in Experiments 1 and 2 was no longer present (Figure B), nor was the relationship between gaze structure and memory performance (Figure C). Likewise, no reliable sequential eye-movement pattern was observed during either the encoding or maintenance period (Figure D and E).

Taken together, these findings suggest that overt eye movements are not merely an incidental correlate of successful performance but make a functional contribution to the organization of sequential memory representations in this task.

At the same time, we agree with the reviewer that these findings do not imply that overt eye movements are the sole mechanism underlying memory organization. Covert attentional processes could also organize memory representations sequentially, and the present experiment was not designed to dissociate their contribution from that of overt eye movements. We have therefore revised the manuscript accordingly and now interpret overt eye movements as one functionally relevant component of a broader organizational process, while highlighting the dissociation between overt and covert sequential mechanisms as an important direction for future work.

We also agree that combining EEG/MEG with approaches such as TDLM would provide a valuable avenue for tracking internally generated sequential representations and clarifying how they relate to overt/covert eye movements. We have added this as an important future direction in the revised Discussion.

We have revised the manuscript accordingly in Results (Pages 11–12), Discussion (Page 17–18) and Methods (Page 22–24).

Minor concerns

The description of spatial frequencies appears confusing, is the period described, which would be in degrees visual angle, or the number of cycles per degree? (methods)

Sorry for the confusion. There are six possible spatial frequencies (0.6, 0.9, 1.3, 1.9, 2.7, and 4 cycles per visual degree, logarithmically spaced). We have modified (Page 22).

Figure 2a right, the y axis is described in the legend as gaze distance to each item. But it is largest when at a specific item? How can distance to something be negative? I think I get the idea behind the figure, but do not understand the data presented.

We thank the reviewer for pointing this out, and we apologize for the lack of clarity. The reviewer is correct that the plotted quantity is not the raw Euclidean distance itself, but a normalized measure of the relative gaze bias toward each item. Specifically, at each time point, we first computed the Euclidean distance between the gaze position and each item location. Because a shorter distance indicates a stronger gaze bias toward that item, we then transformed these distances by mean-centering them across the three items, normalizing by the mean distance, and reversing the sign so that larger values correspond to a stronger relative gaze bias. Consequently, negative values do not indicate a negative distance; rather, they indicate that the gaze was farther from that item relative to the other items at that time point.

We have clarified it in Methods (Page 26 ).

Figure 3b, there is no colour bar label, and I understand that overt replay would not require visiting the precise location, but in this figure, most of the first saccades appear to be within a degree of the fixation point. Perhaps a minimum saccade amplitude might help with this analysis? It occurs to me that those cycling through the objects in the maintenance period might go back to either where they just were, or to the next object in their sequence, and this could be stronger for those struggling with maintenance.

We thank the reviewer for these comments. We have added a label to the color bar in Figure 3B to improve clarity.

We also appreciate the reviewer's observation that many of the first saccades remain relatively close to the fixation point. In this analysis, however, our goal was to characterize the initial orienting response following the retro-cue rather than to determine whether the first saccade landed precisely on a particular item location. Accordingly, we quantified which item location the landing position of the first saccade was closest to, rather than requiring large-amplitude saccades to predefined targets. For this reason, we were hesitant to introduce an additional post hoc saccade-amplitude threshold. Such a criterion would be somewhat arbitrary and could exclude valid cue-related orienting responses.

If we understand the reviewer's final point correctly, the suggestion is that maintenance-related sampling may involve relatively small gaze shifts between relevant item locations rather than precise visits to each location. We agree that this is an interesting possibility and, in fact, it is well aligned with our analytical approach. Both the trajectory-similarity and sequenceness analyses are based on the continuous relative distance between gaze position and each item location, making them sensitive to gradual shifts in gaze bias toward one item relative to the others rather than requiring large or precise object-to-object saccades.

We have clarified this point in the figure legend to avoid the impression that Figure 3B requires precise landing on the cued location (Page 11).

There is a 'value' attributed to the ordinal hierarchy. This is confusing, as there is no inherent value to the objects. There appears to be a salience ranking, which might be worth exploring.

We thank the reviewer for this point. We agree that the term “value” was potentially misleading in the present context because the stimuli did not possess any inherent reward or subjective value. Our intention was simply to refer to the latent ordinal structure defined by the relative spatial frequencies of the stimuli. To avoid confusion, we have removed the term “value” throughout the manuscript and replaced it with terminology that more directly describes the ordinal structure of the stimulus set.

We also agree that low-level visual salience represents an important alternative interpretation. As discussed in our response above, we have revised the Discussion to explicitly consider this possibility and clarify why we believe salience may contribute to the initial orienting of gaze but is unlikely to fully explain the observed sequential organization.

Urban, F., Follet, B., Chamaret, C., Le Meur, O., Baccino, T., & Medium, T. B. (2011). Medium Spatial Frequencies, a Strong Predictor of Salience. Cognitive Computation, 3, 37-47. https://doi.org/10.1007/s12559-010-9086-8ï

Reviewer #3: In two experiments, the authors investigated whether gaze patterns produced during the encoding and maintenance of visual features correlate with the mnemonic organisation of those features. In their study, Gabor patches, encircled by coloured rings varying in green-blue hues, were arranged in either an equilateral triangle or a square configuration. The patches differed in spatial frequency (low, medium, high), which systematically covaried with the colour of the enclosing ring—lower spatial frequencies were more green, whereas higher frequencies were less so. Participants were allowed to freely move their eyes during the memory task, and performance was assessed based on their ability to recall either the location of a probed spatial frequency or colour. The authors analysed gaze patterns during both encoding and maintenance to determine whether eye movements followed the underlying stimulus structure, and report evidence for spontaneous, unprompted sequentialsampling. Based on these findings, they argue that eye movements organise working memory.

This is an interesting study that contributes to our understanding of how sequential sampling may scaffold working memory. The use of a free-viewing task with an embedded stimulus structure that participants can potentially exploit is a compelling approach to examining whether and how behaviour is adapted to optimise memory performance. The study adds to the growing literature linking eye movements and visual working memory. Despite my overall positive impression, I have several major concerns outlined below.

We thank the reviewer for the thoughtful assessment of our manuscript. We have carefully considered each of the concerns raised and have revised the manuscript accordingly. Please see our point-by-point responses below.

1. Most of the conclusions rely on correlational analyses, yet the authors often imply a causal relationship—namely, that eye movements organise visual memory. In essence, the authors correlate the sequence of gaze positions across different stimulus configurations, and then relate differences in this correlation metric to task performance, showing a memory advantage for participants whose gaze follows the hypothesised sampling strategy. However, this relationship could equally be interpreted in the opposite direction: that memory strategies guide gaze behaviour. To support a causal claim that eye movements structure working memory—or that differences between high and low performers are driven by gaze order—it would be necessary to include a manipulation of eye movements (e.g., by constraining or scrambling scan paths) and assess its impact on memory performance.

We thank the reviewer for this important comment. We agree that the correlational findings from the main experiment, on their own, do not establish that eye movements causally organize visual working memory. As the reviewer points out, the observed relationship could equally reflect the opposite direction of influence, whereby an underlying memory strategy guides gaze behavior, rather than gaze itself organizing memory.

To address this issue more directly, we conducted a new control experiment in which participants performed the same 3-item task while maintaining central fixation throughout both the encoding and maintenance periods (shown below). By experimentally constraining overt eye movements, this manipulation allowed us to directly test whether preventing spontaneous gaze sampling influences memory performance and the associated sequential organization.

Thirty-seven participants completed this control experiment. After excluding participants who did not maintain central fixation sufficiently, the final sample comprised 31 participants (see supplementary figure 8). Compared with the free-viewing condition (Experiment 1), memory performance was significantly reduced under the fixation constraint (Figure A, t _(75)_ = 4.37, p < 0.001, Cohen’s d = 1.01). In addition, the configuration-specific pattern-similarity effect observed in Experiments 1 and 2 was no longer present (Figure B), nor was the relationship between gaze structure and memory performance (Figure C). Likewise, no reliable sequential eye-movement pattern was observed during either the encoding or maintenance period (Figure D and E).

Taken together, these findings provide more direct evidence that overt eye movements make a functional contribution to memory organization in this task, rather than merely reflecting individual differences in memory strategy. Accordingly, we have revised the manuscript throughout to adopt more cautious claims when interpreting the functional role of overt eye movements (Pages 11–12, Page 17–18 and Page 22–24).

2. More generally, the analytical approach transforms eye movements into a metric that is difficult to interpret in terms of effect size or behavioural relevance. Different classes of eye movements (e.g., fixational movements, large saccades) and their perceptual consequences are well characterised. In the present study, however, gaze is treated in a relatively abstract manner, and the reliance on correlational metrics obscures how eye movements actually unfolded within a trial. As a result, it is difficult to relate the findings to established knowledge about eye movement behaviour.

We thank the reviewer for this important point. We agree that relating the correlation-based analyses to more conventional oculomotor measures makes the results easier to interpret and better connects them to the broader eye-movement literature. Accordingly, we added analyses of standard eye-movement measures, including saccade count, fixation count, saccade amplitude, fixation duration, and the ordinal sequence of fixations during both encoding and maintenance.

As shown below, participants made more saccades in the 4-item task than in the 3-item task during both encoding (Figure A, t _(84)_ = 6.94 , p < 0.001, Cohen’s d = 1.50) and maintenance (Figure E, t_(84)_ = 2.58, p = 0.01, Cohen’s d = 0.56). Fixation counts showed a similar pattern during both encoding (Figure B, t _(84)_ = 7.19 , p < 0.001, Cohen’s d = 1.55) and maintenance (Figure F, t _(84)_ = 3.34, p = 0.001, Cohen’s d = 0.72). Saccade amplitude showed a marginally significant difference between tasks during encoding (Figure C, t _(84)_ = 1.92, p = 0.06, Cohen’s d = 0.42), but not during maintenance (Figure G, t _(84)_ = 1.42, p = 0.16, Cohen’s d = 0.31). Fixation duration did not differ significantly between tasks during either encoding (Figure D, t _(84)_ = 0.91, p = 0.36, Cohen’s d = 0.20) or maintenance (Figure H, t _(84)_ = 0.27, p = 0.79, Cohen’s d = 0.06). Together, these results indicate that the higher memory load in the 4-item task was primarily associated with an increase in the number of eye movements rather than robust changes in saccade amplitude or fixation duration.

To further illustrate how eye movements unfolded within individual trials, we also examined whether fixation sequences followed the ordinal structure of the memorized items. In the 3-item task, fixation order during encoding showed evidence of a 1-2-3 sequence (chance level: trial number = 36, t _(45)_ = 2.76, p = 0.008, Cohen’s d = 0.41; Figure I, left). Importantly, this sequential fixation pattern was also associated with behavioral performance (Figure I, right): it was significant in high-performing participants (t _(22)_ = 3.40, p = 0.003, Cohen’s d = 0.71), but not in low-performing participants (t _(22)_ = 0.28, p = 0.78, Cohen’s d = 0.06). A similar pattern was observed in the 4-item task (Figure J), where high-performing participants showed evidence of a 1–2–3–4 fixation sequence during encoding (chance level: trial number = 18, high-performing: t _(19)_ = 2.31, p = 0.03, Cohen’s d = 0.52; low-performing: t _(19)_ = –1.39, p = 0.18, Cohen’s d = –0.31), despite the absence of a significant group-level effect (t _(39)_ = 1.51, p = 0.14, Cohen’s d = 0.24).

During the maintenance period, no reliable fixation sequence was detected (Figure I&J, left), likely because many trials contained few overt saccades and fixations (Figure E&F), limiting the extent to which a complete sequential ordinal scan path could be expressed.

At the same time, we believe the continuous gaze-distance analysis provides complementary information that is not fully captured by event-based measures alone. Because overt eye movements during maintenance were relatively sparse, analyses based solely on discrete saccades and fixations may fail to capture more subtle changes in gaze bias. By quantifying gaze continuously over time, the distance-based analysis is able to capture the evolving sequential organization of gaze even when only a few overt saccades or fixations occur. Together, these additional analyses provide a more conventional characterization of eye-movement behavior while complementing the continuous gaze-distance analyses presented in the main manuscript.

These new analyses have been added as supplementary figure 7 and described in the revised manuscript (Pages 8 and Page 26).

3. It is also unclear why the use of two features (colour and spatial frequency) is necessary to induce a latent structure in the stimuli. Is there a reason to believe that a single feature dimension would not suffice to produce an ordering effect reflected in gaze behaviour? Clarifying this design choice would strengthen the rationale of the study.

We thank the reviewer for this thoughtful question. We agree that, in principle, a single feature dimension could be sufficient to induce an ordering effect. Our intention was not to suggest that two feature dimensions are strictly necessary for sequential sampling to emerge. Rather, we used two feature dimensions that varied in a consistent ordinal manner because this creates a richer latent relational structure than either feature alone.

Specifically, the shared ordinal relationship is expressed simultaneously across both color and spatial frequency, encouraging participants to represent the stimulus set in terms of its underlying structure rather than as two independent feature dimensions. In this sense, the latent structure is not tied to a particular feature, but to the ordinal relationship shared across features.

We reasoned that such a representation would make relational encoding and ordinal sampling more advantageous than memorizing each feature independently, thereby increasing the likelihood that spontaneous structure-based sampling strategies would be expressed in gaze behavior.

We have clarified this design rationale in the revised manuscript (Pages 22–23).

4. If eye movements indeed support the organisation of working memory, it is unclear why within-pattern correlations disappear in Experiment 2. Does this suggest that participants no longer follow the prescribed stimulus structure? If the structure re-emerges during maintenance, does this imply that it was learned during encoding despite gaze being misaligned with the expected trajectories? If so, this would suggest that the structure can be acquired without structured eye movements, which weakens the argument for active sensing. The authors attribute the difference between Experiments 1 and 2 to task complexity, but this explanation is not entirely convincing. If anything, one might expect more efficient encoding strategies—particularly among high performers—under higher load. This issue warrants further discussion.

We thank the reviewer for this important and thoughtful comment. We agree that the difference between Experiments 1 and 2 requires a more careful interpretation. Importantly, however, the within-pattern effect did not disappear entirely in Experiment 2. As shown in Figure 5C and 5E, trials sharing the same configuration pattern still showed greater trajectory similarity than trials drawn from different configurations during both encoding and maintenance. This indicates that participants remained sensitive to the underlying ordinal structure even in the 4-item task.

At the same time, the sequenceness analysis revealed a weaker and less consistent ordinal sampling pattern during encoding (Figure 5G). We think this apparent discrepancy reflects the fact that the two analyses capture different aspects of gaze behavior. The pattern-similarity analysis asks whether participants express a similar organizational strategy across trials, whereas the sequenceness analysis additionally requires a stable ordered trajectory to be expressed over time. In this sense, the sequenceness analysis represents a more stringent test of structured sampling.

We therefore do not interpret the absence of a robust encoding sequenceness effect as evidence that the latent structure was not acquired. Rather, it suggests that under the higher memory demands of the 4-item task, participants expressed the latent structure less consistently in overt gaze during encoding. The re-emergence of structured sampling during maintenance further suggests that participants reorganized or reinforced the memorized items according to the latent ordinal structure after all items had been encoded.

We also agree that increasing task demands could, in principle, encourage participants to adopt more efficient encoding strategies. However, a stronger incentive to encode efficiently does not necessarily imply that such strategies can be implemented equally successfully under higher memory load. We therefore think that the 4-item task increased both the need for structured encoding and the difficulty of expressing a stable multi-step sampling sequence online. Under these conditions, successful performance may depend more strongly on subsequent reorganization during the maintenance period.

Taken together, we interpret the results of Experiment 2 as indicating that latent structure can still be acquired under higher memory load even when a stable ordinal eye-movement sequence is not consistently expressed during encoding. We have therefore revised the manuscript to avoid implying that structured eye movements are strictly necessary for structure acquisition. Instead, we argue more cautiously that overt eye movements contribute to the organization and reorganization of structured working-memory representations, with this contribution becoming particularly evident during the maintenance period under higher memory load.

We have accordingly revised both the Results (Page 14) and the Discussion (Page 18) to clarify this interpretation.

5. It would be informative to examine whether low- and high-performing participants differ in basic eye movement parameters across both experiments. For example, how precisely did saccades land on the stimuli? How consistent were saccade amplitudes within and across participants? How long did participants fixate each stimulus before initiating the next saccade? Such analyses could provide more mechanistic insight into the observed effects.

We thank the reviewer for this point. As suggested, we examined whether low- and high-performing participants differed in several standard eye-movement measures , including saccade count, saccade amplitude, and fixation duration. We did not observe significant group differences in any of these measures in Experiment 1 (saccade count, encoding period: t _(44)_ = 0.55, p = 0.59, Cohen’s d = 0.16; maintenance period: t _(44)_ = –1.37, p = 0.18, Cohen’s d = –0.41; saccade amplitude, encoding peirod: t _(44)_ = –0.12, p = 0.91, Cohen’s d = –0.03; maintenance period: t _(44)_ = –0.53, p = 0.60, Cohen’s d = –0.16; fixation duration, encoding peirod: t _(44)_ = –0.42, p = 0.68, Cohen’s d = –0.12; maintenance period: t _(44)_ = 1.52, p = 0.14, Cohen’s d = 0.45).

A similar pattern was observed in Experiment 2. The only significant difference was that high-performing participants made more saccades during encoding than low-performing participants (t _(38)_ = 2.41, p = 0.02, Cohen's d = 0.76). All other comparisons in Experiment 2 were not significant (saccade count, maintenance period: t _(38)_ = –0.14, p = 0.89, Cohen’s d = –0.05; saccade amplitude, encoding peirod: t _(38)_ = –0.43, p = 0.67, Cohen’s d = –0.14; maintenance period: t _(38)_ = 0.22, p = 0.82, Cohen’s d = 0.07; fixation duration, encoding peirod: t _(38)_ = –1.22, p = 0.23, Cohen’s d = 0.39; maintenance period: t _(38)_ = 0.31, p = 0.76, Cohen’s d = 0.10).

Taken together, these analyses suggest that high- and low-performing participants did not differ systematically in the basic characteristics of individual eye movements, such as how often or how far they moved their eyes, or how long they fixated each item. Instead, the difference lay in the sequential organization of those eye movements. This finding further supports the idea that the behavioral advantage observed in high-performing participants is more closely related to how gaze was organized across multiple items based on the underlying ordinal structure than to basic oculomotor performance itself.

6. It would also be helpful to report overall memory performance separately for colour and spatial frequency probes, as this could reveal potential asymmetries between feature dimensions.

We thank the reviewer for this helpful suggestion. We have now reported memory performance separately for colour and spatial-frequency probes. In the 3-item task, performance was significantly above chance for both colour probes (t _(45)_ = 22.87, p < 0.001, Cohen’s d = 3.37) and spatial-frequency probes (t _(45)_ = 15.50, p < 0.001, Cohen’s d = 2.29). Similarly, in the 4-item task, performance was significantly above chance for both colour probes (t _(39)_ = 21.05, p < 0.001, Cohen’s d = 3.33) and spatial-frequency probes (t _(39)_ = 17.15, p < 0.001, Cohen’s d = 2.71).

We also directly compared performance between the two feature dimensions. In the 3-item task, memory performance was significantly higher for colour than for spatial frequency (t _(45)_ = 3.41, p = 0.001, Cohen’s d = 0.50). In the 4-item task, the same trend was present (t _(39)_ = 1.73, p = 0.09, Cohen’s d = 0.27). Thus, the data suggest an advantage for colour over spatial frequency, particularly in the 3-item task.

We have added these analyses to the revised manuscript (Pages 5 and 13). At present, we do not wish to over-interpret the source of this asymmetry. One possible explanation is that colour information may be more readily categorized or verbally labeled than spatial frequency, thereby facilitating memory performance. However, the present study was not designed to investigate feature-specific encoding strategies, and we therefore consider this interpretation speculative.

7. An interesting aspect of the findings is the reported correlation between gaze behaviour and memory performance. This stands in contrast to work from the van Ede lab, which has demonstrated spatial gaze biases during memory tasks that do not predict performance. It would be valuable for the authors to discuss why their task reveals a performance relationship whereas previous studies have not.

We thank the reviewer for this insightful comment. We agree that it is important to discuss why the present study revealed a relationship between gaze behavior and memory performance, whereas work from the van Ede lab has generally interpreted spatial gaze biases as markers of internal attentional selection without a corresponding relationship to behavioral performance.

We think this difference likely reflects the distinct aspects of memory captured by the gaze measures in the two paradigms. In studies such as “*Human gaze tracks attentional focusing in memorized visual space”* and “*Goal-directed and stimulus-driven selection of internal representations”,* gaze biases primarily index the internal selection or prioritization of a single memorized location or representation. In this context, the gaze signal serves mainly as an indicator of which internal representation is currently in the focus of attention. By contrast, the present study focuses on the sequential organization of multiple memorized items. Rather than asking whether gaze is biased toward one remembered location, our analyses quantify whether gaze follows a structured sequential sampling pattern consistent with the latent ordinal organization of the memory set.

We therefore think it is plausible that these two classes of gaze measures differ in their relationship to behavior. A gaze bias toward a single remembered location may primarily reflect the current focus of internal attention, whereas the sequential sampling patterns examined here are more directly related to how multiple memory representations are organized and reorganized over time. Because successful performance in our task depends on leveraging the relational structure across several items to organize memory, individual differences in sequential organization may be expected to have a stronger relationship with memory performance.

At the same time, we do not view these findings as contradictory. Rather, we think they are complementary and suggest that eye movements can reflect multiple cognitive processes during working memory, including both the selection of individual representations and the sequential organization of multiple representations.

We have revised the Discussion accordingly to make this distinction more explicit (Page 20).

8. Finally, over the past decade, a substantial body of work has established a close link between eye movements and visual working memory. The present paper would benefit from more thoroughly situating its findings within this existing literature. For instance:

Heuer, A., Ohl, S., & Rolfs, M. (2020). Memory for action: A functional view of selection in visual working memory. Visual Cognition, 28(5-8), 388-400. https://doi.org/10.1080/13506285.2020.1764156

Ohl, S., & Rolfs, M. (2017). Saccadic eye movements impose a natural bottleneck on visual short-term memory. Journal of Experimental Psychology: Learning, Memory, and Cognition, 43(5), 736-748.

Ohl, S., & Rolfs, M. (2024). Saccadic selection in visual working memory is robust across the visual field and linked to saccade metrics: Evidence from nine experiments and more than 100,000 trials. Journal of Experimental Psychology General, 153(2), 544-563.

Olivers, C. N. L., & Roelfsema, P. R. (2020). Attention for action in visual working memory. Cortex, 131, 179-194. https://doi.org/10.1016/j.cortex.2020.07.011

Schut, M. J., Van der Stoep, N., Postma, A., & Van der Stigchel, S. (2017). The cost of making an eye movement: A direct link between visual working memory and saccade execution. Journal of Vision, 17(6), 15. https://doi.org/10.1167/17.6.15

Shao, N., Li, J., Shui, R., Zheng, X., Lu, J., & Shen, M. (2010). Saccades elicit obligatory allocation of visual working memory. Memory & Cognition, 38(5), 629-640. https://doi.org/10.3758/MC.38.5.629

van der Stigchel, S., & Hollingworth, A. (2018). Visuospatial Working Memory as a Fundamental Component of the Eye Movement System. Current Directions in Psychological Science, 27(2), 136-143. https://doi.org/10.1177/0963721417741710

van Ede, F. (2020). Visual working memory and action: Functional links and bi-directional influences. Visual Cognition, 28(5-8), 401-413. https://doi.org/10.1080/13506285.2020.1759744

van Ede, F., Board, A. G., & Nobre, A. C. (2020). Goal-directed and stimulus-driven selection of internal representations. Proceedings of the National Academy of Sciences, 117(39), 24590-24598. https://doi.org/10.1073/pnas.2013432117

van Ede, F., & Nobre, A. C. (2022). Turning attention inside out: How working memory serves behavior. Nature Reviews Psychology.

van Loon, A. M., Olmos-Solis, K., & Olivers, C. N. L. (2017). Subtle eye movement metrics reveal task-relevant representations prior to visual search. Journal of Vision, 17(6), 1-15. https://doi.org/10.1167/17.6.13

We thank the reviewer for this suggestion. We agree that the manuscript would benefit from being more thoroughly situated within the broader literature linking eye movements and visual working memory, and we have revised the Discussion accordingly (Page 19 and 20). More specifically, the cited work collectively shows that visuospatial working memory is tightly linked to the oculomotor system, with bidirectional influences between internal representations, attentional prioritization, planning and execution (e.g., Shao et al., 2010; Schut et al., 2017; van der Stigchel & Hollingworth, 2018; Heuer et al., 2020; Olivers & Roelfsema, 2020; van Ede, 2020; van Ede & Nobre, 2023; Ohl & Rolfs, 2017, 2024; van Loon et al., 2017). We now make clearer that the present study builds on this literature by asking not only whether gaze reflects internal memory states, but also whether eye movements may contribute to memory organization by following a latent ordinal structure across multiple memorized items, and how this structured organization changes across encoding, maintenance, and task demand.

Reviewer #4: This paper shows that when people hold multiple items in working memory, their eye movements spontaneously follow a hidden ordinal structure linking color and spatial frequency, and stronger structure during encoding predicts better memory. In the easier 3-item task, high performers expressed this ordered sequence mainly during encoding, while lower performers showed more replay-like revisitation during maintenance; after a retro-cue, subsequent saccades also followed the same order. In a harder 4-item task, encoding-based organization weakened and structured replay shifted toward maintenance, supporting the claim that eye movements adaptively help organize memory when cognitive demand is high.

We thank the reviewer for the thoughtful comments of our manuscript. We have carefully considered each of the concerns raised and have revised the manuscript accordingly. Please see our point-by-point responses below.

1. The latent ordinal relationship is implicit but impacts performance. It's unclear when participants pick up on the implicit organization. Since correlations with performance and median split analyses were conducted, it would be useful to know whether this is related to a learning effect or whether it holds across all trials.

We thank the reviewer for this helpful suggestion. To examine whether the observed memory organization based on the latent ordinal structure reflects a gradual learning effect or is already present throughout the experiment, we divided the experiment into early and late halves and compared both memory performance and gaze-based measures across these two phases.

Memory performance was significantly higher in the late than in the early half for both tasks (3-item: t _(45)_ = 4.57, p < 0.001, Cohen’s d = 0.67, Figure A); 4-item: t _(39)_ = 5.03, p < 0.001, Cohen’s d = 0.80, Figure G), indicating some improvement over the course of the experiment.

Importantly, however, the configuration-specific pattern-similarity effect was already present in the early half of the experiment (Figures B and H). In both the 3-item and 4-item tasks, trials drawn from the same configuration showed greater trajectory similarity than trials drawn from different configurations during both encoding and maintenance (3-item, encoding period: t _(45)_ = 7.13, p < 0.001, Cohen’s d = 1.05, maintenance period: t _(45)_ =2.73, p = 0.009, Cohen’s d = 0.40; 4-item: encoding period: t _(39)_ = 10.00, p < 0.001, Cohen’s d = 1.58, maintenance period: t _(39)_ = 4.35, p < 0.001, Cohen’s d = 0.69). Moreover, this effect did not differ reliably between the early and late halves in either experiment (3-item, encoding period: t _(45)_ = –0.01, p = 0.99, Cohen’s d = –0.002, maintenance period: t _(45)_ = 0.41, p = 0.68, Cohen’s d = 0.06; 4-item, encoding period: t _(39)_ = 0.20, p = 0.84, Cohen’s d = 0.03), , with the exception of a marginally weaker maintenance effect in the late half of the 4-item task (t _(39)_ = –1.92, p = 0.06, Cohen’s d = –0.30). These findings suggest that participants were already sensitive to the latent organization relatively early in the experiment, rather than acquiring it only after extended exposure. This interpretation is also consistent with our design rationale, namely to make the underlying structure sufficiently obvious that participants could detect and use it readily.

At the same time, it is important to note that the pattern-similarity analysis reflects sensitivity to the latent ordinal structure but does not by itself require a stable 1–2–3 or 1–2–3–4 sequential sampling pattern. Because the sequenceness analysis provides a more stringent test of stable ordered sampling, we also examined it separately for the early and late halves of the experiment (Figure CDEF for 3-item task and Figure IJKL for 4-item task). At a descriptive level, the 1–2–3 encoding sequence in the 3-item task was already evident as a trend in the early half, particularly among high-performing participants, and became more pronounced in the late half. Likewise, in the 4-item task, the 1–2–3–4 sequential revisitation pattern during maintenance appeared more clearly in the late half, again particularly among high-performing participants. Meanwhile, we note that these comparisons should be interpreted cautiously, because splitting the data substantially reduced statistical power.

Taken together, these findings suggest that participants became sensitive to the latent ordinal structure relatively early in the experiment, while the more stable sequential sampling strategy showed some evidence of becoming more consistently expressed with experience across trials. Thus, the data are consistent with an early acquisition of the latent structure, together with some subsequent refinement of the sequential sampling strategy over time.

We have incorporated this into Results section (Page 6 & 7) and reported these results in supplementary figure 4 .

2. Were participants asked about whether they picked up on the stimulus structure? I think there is a distinction between a higher-order cognitive strategy derived from explicit knowledge of task structure and one that is more automatic or implicitly learned.

We thank the reviewer for this interesting point. During data collection, we informally asked several participants whether they had noticed any regularities in the stimulus set. Some reported that they had become aware of the consistent changes in color and spatial frequency and felt that this regularity helped them remember the stimuli more effectively. However, these observations were collected only informally and were not systematically assessed or recorded across participants. We therefore do not consider them to constitute formal evidence regarding explicit awareness in the present study.

We agree with the reviewer that an important distinction may exist between a higher-order cognitive strategy based on explicit awareness of the task structure and a more automatic or implicitly acquired form of structured sampling. Our study was not designed to distinguish between these possibilities. Addressing this question will require future studies that directly assess participants' awareness, for example, through post-experiment questionnaires or explicit knowledge tests.

3. The observation that eye-movement reinstatement is modulated by cognitive load has been shown by Wynn et al. (2018), see: "Wynn (2018). Fixation reinstatement supports visuospatial memory in older adults. Journal of Experimental Psychology: Human Perception and Performance, 44(7), 1119-1127.". This is relevant to the 3 vs 4 item load changes as a function of overall performance.

We thank the reviewer for pointing out this relevant study. Wynn et al. (2018) is highly related to our findings, particularly in showing that fixation reinstatement can vary as a function of cognitive load and may reflect a compensatory response that supports visuospatial memory performance. This aligns well with our interpretation of the differences between the 3-item and 4-item tasks, in which increasing memory load was associated with a shift in the expression of structured eye-movement organization from encoding to the maintenance period. We have now added this reference and incorporated it into the revised Discussion to better situate our findings within the existing literature (Page 18).

4. The median-split analysis is easy to visualize, but because it discards information and imposes an arbitrary threshold, the main claims about performance-dependent encoding versus maintenance effects would be stronger if supplemented with an analysis treating performance continuously, ideally in a mixed-effects framework (or a lag-window summary analyzed continuously).

We thank the reviewer for this important point. Following the reviewer’s suggestion, we supplemented the median-split analysis with continuous participant-level regression analyses, conducted separately for the 3-item and 4-item tasks, to test whether overall memory performance was predicted by the encoding- and maintenance-period sequence scores.

In the 3-item task, the regression model was significant (F_(2,43)_ = 5.07, p = 0.011, R^2^ = 0.19). Importantly, only the maintenance-period sequence score significantly predicted memory performance (b = –0.134, t = –3.10, p=0.003), whereas the encoding-period sequence score did not (b=0.02, t=0.58, p=0.57). In the 4-item task, the model was also significant (F_(2,37)_=4.19), p=0.02, R^2^=0.18). Again, only the maintenance-period sequence score significantly predicted memory performance (b = 0.15, t = 2.56, p=0.01), whereas the encoding-period sequence score did not (b = –0.01, t = –0.34, p=0.73).

Overall, these continuous analyses primarily supported the maintenance-related effects observed in the median-split analyses. Specifically, in the 3-item task, the negative relationship between maintenance-period sequence strength and memory performance is in line with the median-split result showing that low-performing participants, but not high-performing participants, exhibited sequential revisitation during maintenance. In the 4-item task, the positive relationship between maintenance-period sequence strength and memory performance is consistent with the median-split finding that maintenance-period sequential eye movements were only observed in high-performing participants.

At the same time, the continuous analyses did not reveal a reliable relationship between encoding-period sequence strength and memory performance in either task. One possible explanation is that encoding-related sequence expression may be less strongly differentiated across individuals, whereas maintenance-related sequence expression may more directly reflect variability in post-encoding reorganization across participants.

We note, however, that this continuous analysis necessarily depends on how the sequence score is summarized across time. In the present study, sequence scores were defined by averaging sequence strength across lag windows that exceeded the permutation threshold in the sequenceness analysis. For the maintenance period, these lag windows were defined based on subgroup-level effects, because no significant lag window was present in the full sample. Accordingly, we regard the continuous regression as a complementary, albeit somewhat data-driven, summary analysis rather than as a fully independent confirmatory test of the median-split results.
