# Supplementary material for: Eye movements reflect capacity-dependent organization of working memory
Source: PLoS Biol. 2026 Oct 13;24(10):e3004047. doi: 10.1371/journal.pbio.3004047 (PMC13674860; doi:10.1371/journal.pbio.3004047)
Supplement: Supplementary file 3 [file pbio.3004047.s012.docx]

Responses to Editorial Requests

1 - ETHICS STATEMENT: Please update the ethics statement in your materials and methods section, to indicate whether the study was conducted in accordance with the principles expressed in the Declaration of Helsinki.

We thank the Editors for this request. We have added the ethics statement in the Methods section to clarify that the study was conducted in accordance with the principles expressed in the Declaration of Helsinki.

2 - DATA: Thank you for providing the underlying data for your study on OSF. Please update this deposition to include a 'readme' file (or description) detailing what the data are and how they relate to the figures presented in your study. Please also update all the figure legends pointing readers to the underlying data.

We have updated our OSF repository to include a description of the underlying data, their organization, and how they relate to the analyses and figures presented in the manuscript. We have also updated the figure legends to indicate where the underlying data can be accessed.

3 - CODE: Per journal policy, if you have generated any custom code during the course of this investigation, please make it available without restrictions. Please ensure that the code is sufficiently well documented and reusable, and that your Data Statement in the Editorial Manager submission system accurately describes where your code can be found. More information on our Code Policy, what and how to share can be found here: https://journals.plos.org/plosbiology/s/code-availability

We have made the custom analysis code used for the main analyses available without restriction in our repository. The code includes documentation for the pattern-similarity, sequenceness, and retro-cue saccade-transition analyses. We have also updated the Data Availability Statement in the submission system to provide the location of the analysis code.

----------------------------------------------------------------

REVIEWS:

Reviewer #1, Andrey R. Nikolaev (note, reviewer 1 has signed this review): The authors have responded constructively and substantially strengthened the paper. I have only one remaining point about Experiment 2. Because it had fewer trials per configuration, fewer two-saccade trials, and more possible transitions, the absence of reliable ordered structure may reflect lower sensitivity rather than a change in strategy. The maintenance effect in high performers does not fully resolve this, since sensitivity may differ across phases and subgroups. I therefore suggest that the authors clarify that the load-dependent interpretation remains tentative and that generalization beyond three-item displays is limited. These textual changes would be sufficient, although a trial-matched subsampling analysis (repeating the Experiment 1 analysis with 18 trials per configuration) would be helpful.

I appreciate the authors' addition of the refixation literature but the cited Nikolaev et al. (2025) reference appears to be a different 2025 paper. The intended reference was:

Nikolaev, A. R., Meghanathan, R. N., & Van Leeuwen, C. (2025). Refixation behavior in naturalistic viewing: Methods, mechanisms, and neural correlates. Attention, Perception, & Psychophysics, 87 (1), 25-49.

We thank the reviewer for this thoughtful comment and for suggesting the trial-matched subsampling analysis. We agree that the smaller number of trials per configuration in Experiment 2 could reduce sensitivity for detecting configuration-specific and sequential gaze structure, and that this should be considered when interpreting differences between the 3-item and 4-item tasks.

To address this concern directly, we performed the suggested trial-matched analysis in Experiment 1. For each participant, we randomly subsampled 18 of the 36 trials available for each spatial configuration, thereby matching the number of trials per configuration in Experiment 2, and repeated the analyses on the subsampled data. Importantly, the trial-matched analysis reproduced the same overall pattern observed using all 36 trials per configuration (shown below, added in supplementary figure 9). Thus, reducing Experiment 1 to the same number of trials per configuration as Experiment 2 did not eliminate the structured gaze effects observed in the 3-item task. This suggests that the weaker encoding-related effects in Experiment 2 are unlikely to be explained solely by the smaller number of trials per configuration.

This conclusion is also consistent with our early-versus-late analysis reported in supplementary figure 4. Dividing Experiment 1 into two halves resulted in approximately 18 trials per configuration in each half, providing an additional analysis at a trial count comparable to Experiment 2. During this analysis, we identified and corrected a coding error that affected the previously reported early-versus-late pattern-similarity analysis only. We have updated the corresponding results and supplementary figure 4 accordingly; this correction does not affect the main analyses or conclusions of the study. In the 3-item task, the configuration-specific pattern-similarity effect was present in both the early and late halves during encoding (early: t _(45)_ = 5.43, p < 0.001, Cohen’s d = 0.80; late: t _(45)_ = 7.40, p < 0.001 , Cohen’s d = 1.09). During maintenance, the effect was absent in the early half but emerged in the late half (early: t _(45)_ = 1.34, p = 0.19, Cohen’s d = 0.20; late: t _(45)_ = 2.90, p = 0.006, Cohen’s d = 0.43). In the 4-item task, the pattern-similarity effect was observed in both halves during both encoding (early: t _(39)_ = 8.43, p < 0.001, Cohen’s d = 1.33; late: t _(39)_ = 8.50, p < 0.001, Cohen’s d = 1.34) and maintenance (early: t _(39)_ = 3.03, p = 0.004, Cohen’s d = 0.48; late: t _(39)_ = 2.97, p = 0.005, Cohen’s d = 0.47). A significant difference between the early and late halves was observed only during encoding in the 3-item task (t _(45)_ = 5.23, p < 0.001, Cohen’s d = 0.77), but not during maintenance in the 3-item task (t _(45)_ = 1.33, p = 0.19, Cohen’s d = 0.20), or during encoding or maintenance in the 4-item task (encoding: t _(39)_ = 1.15, p = 0.26, Cohen’s d = 0.18; maintenance period: t _(39)_ = 0.77, p = 0.45, Cohen’s d = 0.12).

Together, these analyses indicate that the differences between Experiments 1 and 2 are unlikely to be explained solely by the smaller number of trials per configuration in Experiment 2. In particular, the structured gaze effects in Experiment 1 remained detectable when the number of trials per configuration was matched to Experiment 2, providing additional support for our interpretation that the differences between the two experiments are related to increased task demands. At the same time, we agree with the reviewer that other differences between the two experiments, including the larger number of possible transitions and fewer trials containing multiple saccades in the 4-item task, may also contribute to differences in sensitivity. We have therefore clarified in the Discussion that the load-dependent interpretation should be considered with these design differences in mind, and that further studies using more closely matched designs will be important for determining how sequential gaze organization generalizes across different memory loads (Page 20).

We also thank the reviewer for identifying the incorrect Nikolaev et al. (2025) reference. We have replaced it with the intended reference: Nikolaev, A. R., Meghanathan, R. N., & Van Leeuwen, C. (2025). Refixation behavior in naturalistic viewing: Methods, mechanisms, and neural correlates. Attention, Perception, & Psychophysics, 87(1), 25–49.

Reviewer #2: General Evaluation

The authors have put a notable amount of work into this revision, and I commend them for addressing the performance comparisons between the experiments. However, several critical theoretical and methodological issues remain unaddressed. The primary concern is that the evidence provided still does not support the strong causal claim in the abstract that "eye movements structure memory." Instead, the data are much more consistent with a softer interpretation: that eye movements reveal an internally generated organizational process.

1. Methodological Control and Causal Claims (Abstract vs. Discussion)

*	Abstract Claim: The abstract retains the strong claim that eye movements structure memory.

*	Experiment 3 Limitations: The authors added Experiment 3 to control for eye movements by asking participants to remain fixated. While two previous reviewers suggested this type of constraint as a control, it introduces a severe confound regarding information acquisition.

*	Information Acquisition vs. Motor Control: As the authors note in the introduction, the central fovea is required to sample different parts of a scene due to its limited visual angle. Forcing central fixation naturally degrades the sensory input of peripheral objects. Therefore, decreased memory performance in this condition is likely driven by poorer initial information acquisition, not the absence of eye movements structuring the memory itself.

*	Lack of Control: Without a control for information acquisition, the comparison between these experiments cannot support the claim that eye movements causally structure memory.

*	Consistency in Manuscript: Interestingly, the discussion has been appropriately softened to state that eye movements "reveal an internally generated organizational process." I fully agree with this framing. The data suggest that an internal mental process directs eye movements, meaning the gaze patterns reveal the underlying structure rather than building it. The abstract must be updated to match this more accurate, softened interpretation.

We thank the reviewer for raising this important concern. We agree that the present findings do not sufficiently support the strong causal claim that “eye movements structure memory.” Rather, as we stated in the Discussion, we believe the findings are more appropriately interpreted as showing that structured eye movements reflect an internally generated organizational process whose expression changes with task demands. We appreciate the reviewer identifying that this more cautious interpretation was not consistently reflected in the Abstract in our previous revision. We have now revised the Abstract accordingly:

*" Human cognition is capacity-limited, requiring strategies to actively structure information. Eye movements offer a natural mechanism for sequential sampling, but whether such sequences reflect memory organization is unknown. We developed a working-memory task where color-frequency pairings created a latent ordinal structure to optimally reduce memory load. Across two experiments, gaze patterns spontaneously aligned with this structure. Participants sampled items following this sequence during encoding and revisited them during maintenance. Critically, the expression of this structure depended on cognitive demand. In a 3-item task, high performers showed robust sequential sampling during encoding, whereas lower performers compensated with replay-like revisitation during maintenance. Under higher demand (4 items), encoding-based organization was disrupted, and structured revisitation emerged primarily during maintenance. These findings suggest that eye movements provide an overt expression of an internally generated organizational process that flexibly adapts to cognitive demands, revealing a behavioral analogue to neural replay when encoding resources are strained."*

We also thank the reviewer for highlighting an important limitation of the newly added Experiment 3 (referred to as the control experiment in the manuscript). This experiment was introduced in response to the previous reviewers’ suggestion to test whether restricting eye movements would impair memory performance. We believe that Experiment 3 remains informative in showing that restricting natural eye movements was associated with reduced memory performance and the disappearance of the structured gaze patterns observed under free viewing. Thus, allowing natural eye movements is associated with both better memory performance and the expression of structured gaze behavior in the present task.

At the same time, we agree with the reviewer that this manipulation does not fully dissociate the consequences of restricting eye movements from those of altered visual information acquisition. Because maintaining central fixation necessarily limits foveal sampling of the peripheral stimuli, the reduction in memory performance cannot be attributed specifically to the absence of eye movements as an organizational mechanism. Experiment 3 therefore does not establish that overt eye movements themselves are necessary for, or causally structure, memory organization.

Accordingly, we now interpret Experiment 3 more narrowly as demonstrating the behavioral consequences associated with restricting natural eye movements, while acknowledging that these consequences may reflect differences in visual information acquisition, memory-related organization, or both.

We have revised the corresponding text in the manuscript to make this limitation explicit (Page 12, 18, 19).

2. Alternative Explanations: Salience vs. Strategic Anchoring

*	Dismissal of Spatial Frequency: The authors dismiss the hypothesis that low spatial frequency salience drives initial encoding sampling (e.g., Urban et al., 2011). They do so based on task differences rather than empirical evidence.

*	Salience Gradient Model: In the absence of data to the contrary, a bottom-up salience model remains a highly parsimonious explanation. Humans naturally look at low-frequency objects first due to salience. Once the lowest frequency object is fixated, the next lowest frequency object becomes the most salient item in the remaining field (accounting for retinal eccentricity and distance discounting; see Donk et al., 2024).

*	Sampling Efficiency: This type of salience gradient or simple sampling efficiency can fully explain why sequential sampling appears in Experiment 1 but changes in Experiment 2 (where diagonal, longer saccades might disrupt the sequence). This mechanism would still lead to higher within-configuration correlations than between-configuration correlations.

*	Anchoring Evidence: The authors' appeal to a "mental number line" to explain anchoring lacks convincing evidence. The manuscript should seriously engage with and discuss the bottom-up salience account as a viable alternative to their top-down anchoring theory.

We thank the reviewer for further clarifying this important alternative account. We agree that bottom-up visual salience provides a viable explanation for at least part of the sequential sampling observed during encoding. Specifically, if items differing in spatial frequency also differed systematically in visual salience, participants might initially orient toward the most salient item and subsequently toward the next most salient item, thereby generating an ordered sampling sequence without requiring a top-down anchoring strategy. Importantly, we did not independently measure or manipulate the visual salience of the individual gratings and therefore cannot determine whether the low-, medium-, and high-frequency items within a trial formed a corresponding salience gradient. We therefore agree that the present data cannot conclusively distinguish a bottom-up salience-based account from a strategic organizational account for the encoding-period sequence.

We nevertheless think it is useful to clarify what is established by the existing spatial-frequency literature. Urban et al. (2011) showed that spatial-frequency content contributes to gaze allocation, with the low-frequency range (0.7–1.3 cycles/degree) providing the strongest overall prediction of fixation locations, although the preferred frequency range varied across scene categories. These findings establish a relationship between spatial frequency and gaze allocation, but do not demonstrate a monotonic salience gradient across spatial frequencies. Moreover, their analysis concerned absolute spatial-frequency bands in natural scenes, whereas the ordinal positions in our task were defined by the relative spatial frequencies of the items within each trial. Nevertheless, as the reviewer points out, a relative salience gradient could potentially emerge within an individual display. Because we did not directly quantify such a gradient, this possibility remains open.

Accordingly, we have revised the Discussion to present bottom-up salience and strategic organization as alternative, and potentially complementary, accounts of the encoding-period sequence. We have also tempered our previous emphasis on mental-number-line-based anchoring. Rather than attributing the initial low-to-high sampling sequence specifically to strategic anchoring, we now acknowledge that bottom-up salience could contribute to both the initial selection and subsequent ordering of fixations.

We also agree that sampling efficiency could contribute to the difference in encoding sequenceness between the 3-item and 4-item tasks. The 4-item display necessarily involved a more complex spatial arrangement, with different transition paths and saccade distances, which could make a consistent sequential trajectory more difficult to express or detect. We therefore do not attribute the weaker encoding sequenceness in Experiment 2 uniquely to increased memory demand. At the same time, this account does not straightforwardly explain the full pattern across task stages. In the 4-item task, weaker sequential organization during encoding was accompanied by stronger configuration-specific organization and the emergence of sequential revisitation during maintenance. Thus, the effect of increasing task demands was not simply a general reduction in structured gaze, but a change in when structured gaze was most strongly expressed.

Importantly, the broader interpretation of our findings does not require the encoding sequence itself to originate exclusively from top-down organization. Structured gaze was also expressed during maintenance, when the stimuli were no longer visible and therefore could not exert a concurrent bottom-up influence on gaze. We therefore interpret the results as consistent with an interaction between perceptual sampling and internally generated memory organization: bottom-up salience may contribute to establishing the sampling sequence during encoding, whereas its subsequent expression during maintenance reflects organization in the absence of concurrent visual input. The changes in the expression of structured gaze across task stages and memory demands are further consistent with flexible internal organization, although the relative contributions of salience, sampling constraints, and memory demand during encoding cannot be fully separated by the present design.

Future studies that directly manipulate or independently quantify stimulus salience will be important for distinguishing these possibilities. We have revised the Discussion accordingly (Page 21-22).

3. Necessity

*	Necessity vs. Revelation: The data show that high performers reorganize objects in memory to reflect ordinal structure. However, there is no evidence demonstrating that eye movements are necessary for this mental reorganization to occur. The authors must carefully distinguish between a behavior that reveals a cognitive state and a behavior that creates it.

We thank the reviewer for emphasizing this important distinction. We agree that the present findings do not establish that overt eye movements are necessary for the internal reorganization of memory representations. Rather, our data show that structured eye movements provide an observable expression of an underlying organizational process that reflects the latent ordinal structure and varies with task demands. We therefore distinguish between eye movements revealing the organization of mnemonic representations and eye movements being required to create that organization.

As discussed in response to Comment 1, although restricting natural eye movements in Experiment 3 was associated with reduced memory performance and the disappearance of structured gaze patterns, this manipulation also altered visual information acquisition and therefore cannot establish that eye movements themselves are necessary for memory organization.

We have therefore revised the manuscript throughout, including the Abstract, Introduction, Results, and Discussion, to avoid language implying that eye movements are necessary for, or causally create, memory organization (see revisions throughout the manuscript, particularly Pages 2, 4, 6–15, 18–19, and 23). Instead, we consistently describe structured eye movements as an overt expression of an underlying organizational process, while acknowledging that the present data cannot determine whether overt gaze shifts contribute causally to the formation of that organization.

4. Methodological and Formatting Clarifications

*	Sequenceness Analysis: The sequenceness analysis remains difficult to follow. The explanation of this pipeline requires further clarification and transparency.

We thank the reviewer for raising this point. We have added a more detailed explanation of the “Sequenceness Analysis” to improve its clarity (Page 8, 30 & 31).

*	Vector Alternative: A more straightforward, transparent analysis approach would be to project the gaze position vector (from central fixation) onto the object position vector. This approach yields high values during object fixation, low values elsewhere, and avoids opaque normalization steps (such as mean-centering across three items and normalizing by the mean distance) that rely on unreported values. I recognize that this could lead fixations past the eccentricity of the objects to have high values. I do think that treating fixations outside of a certain radius the same way they treat blinks would handle this sufficiently.

We thank the reviewer for this helpful suggestion. We agree that projecting the gaze-position vector onto the object-position vector provides an intuitive alternative way of deriving item-specific gaze signals. We have carefully considered this approach. However, we retained our distance-based measure because it directly quantifies gaze proximity to the actual spatial location of each item, which is the aspect of gaze behavior that our analysis was designed to capture.

Specifically, at each time point, we first calculated the Euclidean distance $d_{i}(t)$ between gaze and each item $i$ , and then transformed this into a relative gaze-bias measure:$G_{i}(t) =-\frac{d_{i}(t) -\bar{d}(t)}{\bar{d}(t)}$, where $\bar{d}(t)$ is the mean gaze-to-item distance across all items at that time point. Thus, positive values indicate that gaze is closer to a given item than the average distance to all items, whereas negative values indicate that gaze is relatively farther from that item. Larger values indicate stronger relative gaze bias toward that item. The mean-centering and normalization were used to express gaze proximity to each item relative to the other items within the same display, rather than relying on absolute gaze-to-item distance.

The vector-projection approach and our distance-based approach capture somewhat different aspects of gaze position. The proposed vector projection quantifies the component of gaze displacement along the vector from central fixation to a particular item, whereas our measure directly quantifies two-dimensional spatial proximity to the item's actual location. As the reviewer also notes, an important issue with the projection approach arises when gaze extends beyond the eccentricity of an item. In this case, the projection value can continue to increase even though gaze is moving farther away from the item. For our purpose of quantifying gaze bias toward an item, we would instead expect the signal to be maximal when gaze is closest to the item and to decrease as gaze moves away from it in any direction. The reviewer suggests addressing the projection issue by excluding gaze positions beyond a certain radius. While this is possible, it would require introducing an additional spatial threshold for determining which gaze positions should be retained. The Euclidean-distance measure naturally captures this property without requiring such an additional threshold.

We nevertheless agree with the reviewer that the rationale and interpretation of our original transformation were not described with sufficient transparency. We have therefore substantially revised the Methods to provide the explicit equation, explain the purpose of the mean-centering and normalization, and clarify the interpretation of the resulting values (Page 29).

*	Figure 2a: The right y-axis of Figure 2a still lacks a descriptive text label. It currently only lists units (DVA). However, because the text states this metric undergoes normalization, it should theoretically be expressed in arbitrary units (a.u.). Please clarify or correct this axis.

Thanks for pointing it out. We have also revised the corresponding figure labeling to make clear that the normalized relative gaze-bias measure is dimensionless rather than expressed in degrees of visual angle.

*	Data Reporting: Throughout the manuscript, the authors should report raw accuracy values (and chance-corrected accuracy where appropriate) rather than just reporting the significance of statistical tests. This will greatly improve the transparency and informativeness of the results

Thanks for raising this point. We have reported both the mean and SD of raw accuracy and corrected accuracy values for all experiments (Page 5, 11 & 13 ).

Donk M, van Heusden E, Olivers CNL. Retinal eccentricity modulates saliency-driven but not relevance-driven visual selection. Atten Percept Psychophys. 2024 Jul;86(5):1609-1620. doi: 10.3758/s13414-024-02848-z. Epub 2024 Jan 25. PMID: 38273181; PMCID: PMC11557663.

Reviewer #3: The authors have carefully addressed all the concerns raised in the first round of reviews. I have no further concerns.

We thank the reviewer for their careful evaluation of our revised manuscript and for their positive feedback.
