# Supplementary material for: Eye movements reflect capacity-dependent organization of working memory
Source: PLoS Biol. 2026 Oct 13;24(10):e3004047. doi: 10.1371/journal.pbio.3004047 (PMC13674860; doi:10.1371/journal.pbio.3004047)
Supplement: Peer review history

# Author comment: Author response to Decision Letter 0

**DOI:** 10.1371/journal.pbio.3004047.r001

19 Jul 2026

Attachment Submitted filename: response to review.docx

# Editor report: Decision Letter 0

**DOI:** 10.1371/journal.pbio.3004047.r002

**Authors:** Lucas Smith

24 Jul 2026

Dear Qiaoli,

Thank you for submitting your revised manuscript entitled "Adaptive sequential eye-movement sampling and reorganization under different task demands" for consideration as a Research Article by PLOS Biology.

Your manuscript has now been evaluated by the PLOS Biology editorial staff and I am writing to let you know that we would like to send your submission back to the original reviewers.

However, before we can send your manuscript to reviewers, we need you to complete your submission by providing the metadata that is required for full assessment. To this end, please login to Editorial Manager where you will find the paper in the 'Submissions Needing Revisions' folder on your homepage. Please click 'Revise Submission' from the Action Links and complete all additional questions in the submission questionnaire.

Once your full submission is complete, your paper will undergo a series of checks in preparation for peer review. After your manuscript has passed the checks it will be sent out for review. To provide the metadata for your submission, please Login to Editorial Manager (https://www.editorialmanager.com/pbiology) within two working days, i.e. by Jul 28 2026 11:59PM.

During the process of completing your manuscript submission, you will be invited to opt-in to posting your pre-review manuscript as a bioRxiv preprint. Visit http://journals.plos.org/plosbiology/s/preprints for full details. If you consent to posting your current manuscript as a preprint, please upload a single Preprint PDF.

Feel free to email us at plosbiology@plos.org if you have any queries relating to your submission.

Kind regards,

Luke

Lucas Smith, Ph.D.

Senior Editor

PLOS Biology

lsmith@plos.org

# Author comment: Author response to Decision Letter 1

**DOI:** 10.1371/journal.pbio.3004047.r003

25 Jul 2026

Attachment Submitted filename: response_to_review_auresp_1.docx

# Peer review: Decision Letter 1

**DOI:** 10.1371/journal.pbio.3004047.r004

**Authors:** Lucas Smith

26 Aug 2026

Dear Dr Huang,

Thank you for your patience while we considered your revised manuscript "Adaptive sequential eye-movement sampling and reorganization under different task demands" for consideration as a Research Article at PLOS Biology. Your revised study has now been evaluated by the PLOS Biology editors, the Academic Editor and three of the original reviewers.

The reviews are appended below, and you will see that the reviewers all appreciate the efforts that have gone into this revision. However, both reviewers 1 and 2 have a number of lingering concerns that we think should be addressed before we can consider study for publication. We think that the remaining reviewer concerns can largely be addressed via textual changes, although we would welcome additional analyses to further support your conclusions, if feasible.

In light of the reviews, which you will find at the end of this email, we are pleased to offer you the opportunity to address the remaining points from the reviewers in a revision that we anticipate should not take you very long - however if you need additional time we are also happy to provide an extension. We will then assess your revised manuscript and your response to the reviewers' comments with our Academic Editor aiming to avoid further rounds of peer-review, although we might need to consult with the reviewers, depending on the nature of the revisions.

In addition to addressing the reviewer concerns, we have a number of editorial requests, detailed below. Please address these along with the reviews:

1 - ETHICS STATEMENT: Please update the ethics statement in your materials and methods section, to indicate whether the study was conducted in accordance with the principles expressed in the Declaration of Helsinki.

2 - DATA: Thank you for providing the underlying data for your study on OSF. Please update this deposition to include a 'readme' file (or description) detailing what the data are and how they relate to the figures presented in your study. Please also update all the figure legends pointing readers to the underlying data.

3 - CODE: Per journal policy, if you have generated any custom code during the course of this investigation, please make it available without restrictions. Please ensure that the code is sufficiently well documented and reusable, and that your Data Statement in the Editorial Manager submission system accurately describes where your code can be found. More information on our Code Policy, what and how to share can be found here: https://journals.plos.org/plosbiology/s/code-availability

Please note that we cannot accept sole deposition of code in GitHub, as this could be changed after publication. However, you can archive this version of your publicly available GitHub code to Zenodo. Once you do this, it will generate a DOI number, which you will need to provide in the Data Accessibility Statement (you are welcome to also provide the GitHub access information). See the process for doing this here: https://docs.github.com/en/repositories/archiving-a-github-repository/referencing-and-citing-content

In addition to these revisions, you may need to complete some formatting changes, which you will receive in a follow up email. A member of our team will be in touch with a set of requests shortly. If you do not receive a separate email within a few days, please assume that checks have been completed, and no additional changes are required.

We expect to receive your revised manuscript within 1 month (but, again, we are happy to grant an extension as needed). Please email us (plosbiology@plos.org) if you have any questions or concerns, or would like to request an extension.

At this stage, your manuscript remains formally under active consideration at our journal; please notify us by email if you do not intend to submit a revision so that we withdraw the manuscript.

**IMPORTANT - SUBMITTING YOUR REVISION**

Your revisions should address the specific points made by each reviewer. Please submit the following files along with your revised manuscript:

1. A 'Response to Reviewers' file - this should detail your responses to the editorial requests, present a point-by-point response to all of the reviewers' comments, and indicate the changes made to the manuscript.

*NOTE: In your point-by-point response to the reviewers, please provide the full context of each review. Do not selectively quote paragraphs or sentences to reply to. The entire set of reviewer comments should be present in full and each specific point should be responded to individually.

You should also cite any additional relevant literature that has been published since the original submission and mention any additional citations in your response.

2. In addition to a clean copy of the manuscript, please also upload a 'track-changes' version of your manuscript that specifies the edits made. This should be uploaded as a "Revised Article with Changes Highlighted " file type.

*Revision Checklist*

When you are ready to submit your revised manuscript, please refer to this revision checklist: https://plos.io/Biology_Checklist

To submit a revised version of your manuscript, please go to https://www.editorialmanager.com/pbiology/ and log in as an Author. Click the link labelled 'Submissions Needing Revision' where you will find your submission record.

Please make sure to read the following important policies and guidelines while preparing your revision:

*Published Peer Review*

Please note while forming your response, if your article is accepted, you may have the opportunity to make the peer review history publicly available. The record will include editor decision letters (with reviews) and your responses to reviewer comments. If eligible, we will contact you to opt in or out. Please see here for more details:

https://blogs.plos.org/plos/2019/05/plos-journals-now-open-for-published-peer-review/

*PLOS Data Policy*

Please note that as a condition of publication PLOS' data policy (http://journals.plos.org/plosbiology/s/data-availability) requires that you make available all data used to draw the conclusions arrived at in your manuscript. If you have not already done so, you must include any data used in your manuscript either in appropriate repositories, within the body of the manuscript, or as supporting information (N.B. this includes any numerical values that were used to generate graphs, histograms etc.).

*Protocols deposition*

To enhance the reproducibility of your results, we recommend that if applicable you deposit your laboratory protocols in protocols.io, where a protocol can be assigned its own identifier (DOI) such that it can be cited independently in the future. Additionally, PLOS ONE offers an option for publishing peer-reviewed Lab Protocol articles, which describe protocols hosted on protocols.io. Read more information on sharing protocols at https://plos.org/protocols?utm_medium=editorial-email&utm_source=authorletters&utm_campaign=protocols

Thank you again for your submission to our journal. We hope that our editorial process has been constructive thus far, and we welcome your feedback at any time. Please don't hesitate to contact us if you have any questions or comments.

Sincerely,

Luke

Lucas Smith, Ph.D.

Senior Editor

PLOS Biology

lsmith@plos.org

----------------------------------------------------------------

REVIEWS:

REVIEWS:

Reviewer #1, Andrey R. Nikolaev (note, reviewer 1 has signed this review): The authors have responded constructively and substantially strengthened the paper. I have only one remaining point about Experiment 2. Because it had fewer trials per configuration, fewer two-saccade trials, and more possible transitions, the absence of reliable ordered structure may reflect lower sensitivity rather than a change in strategy. The maintenance effect in high performers does not fully resolve this, since sensitivity may differ across phases and subgroups. I therefore suggest that the authors clarify that the load-dependent interpretation remains tentative and that generalization beyond three-item displays is limited. These textual changes would be sufficient, although a trial-matched subsampling analysis (repeating the Experiment 1 analysis with 18 trials per configuration) would be helpful.

I appreciate the authors' addition of the refixation literature but the cited Nikolaev et al. (2025) reference appears to be a different 2025 paper. The intended reference was:

Nikolaev, A. R., Meghanathan, R. N., & Van Leeuwen, C. (2025). Refixation behavior in naturalistic viewing: Methods, mechanisms, and neural correlates. Attention, Perception, & Psychophysics, 87 (1), 25-49.

Reviewer #2: General Evaluation

The authors have put a notable amount of work into this revision, and I commend them for addressing the performance comparisons between the experiments. However, several critical theoretical and methodological issues remain unaddressed. The primary concern is that the evidence provided still does not support the strong causal claim in the abstract that "eye movements structure memory." Instead, the data are much more consistent with a softer interpretation: that eye movements reveal an internally generated organizational process.

1. Methodological Control and Causal Claims (Abstract vs. Discussion)

*Abstract Claim: The abstract retains the strong claim that eye movements structure memory.

*Experiment 3 Limitations: The authors added Experiment 3 to control for eye movements by asking participants to remain fixated. While two previous reviewers suggested this type of constraint as a control, it introduces a severe confound regarding information acquisition.

*Information Acquisition vs. Motor Control: As the authors note in the introduction, the central fovea is required to sample different parts of a scene due to its limited visual angle. Forcing central fixation naturally degrades the sensory input of peripheral objects. Therefore, decreased memory performance in this condition is likely driven by poorer initial information acquisition, not the absence of eye movements structuring the memory itself.

*Lack of Control: Without a control for information acquisition, the comparison between these experiments cannot support the claim that eye movements causally structure memory.

*Consistency in Manuscript: Interestingly, the discussion has been appropriately softened to state that eye movements "reveal an internally generated organizational process." I fully agree with this framing. The data suggest that an internal mental process directs eye movements, meaning the gaze patterns reveal the underlying structure rather than building it. The abstract must be updated to match this more accurate, softened interpretation.

2. Alternative Explanations: Salience vs. Strategic Anchoring

*Dismissal of Spatial Frequency: The authors dismiss the hypothesis that low spatial frequency salience drives initial encoding sampling (e.g., Urban et al., 2011). They do so based on task differences rather than empirical evidence.

*Salience Gradient Model: In the absence of data to the contrary, a bottom-up salience model remains a highly parsimonious explanation. Humans naturally look at low-frequency objects first due to salience. Once the lowest frequency object is fixated, the next lowest frequency object becomes the most salient item in the remaining field (accounting for retinal eccentricity and distance discounting; see Donk et al., 2024).

*Sampling Efficiency: This type of salience gradient or simple sampling efficiency can fully explain why sequential sampling appears in Experiment 1 but changes in Experiment 2 (where diagonal, longer saccades might disrupt the sequence). This mechanism would still lead to higher within-configuration correlations than between-configuration correlations.

*Anchoring Evidence: The authors' appeal to a "mental number line" to explain anchoring lacks convincing evidence. The manuscript should seriously engage with and discuss the bottom-up salience account as a viable alternative to their top-down anchoring theory.

3. Necessity

*Necessity vs. Revelation: The data show that high performers reorganize objects in memory to reflect ordinal structure. However, there is no evidence demonstrating that eye movements are necessary for this mental reorganization to occur. The authors must carefully distinguish between a behavior that reveals a cognitive state and a behavior that creates it.

4. Methodological and Formatting Clarifications

*Sequenceness Analysis: The sequenceness analysis remains difficult to follow. The explanation of this pipeline requires further clarification and transparency.

*Vector Alternative: A more straightforward, transparent analysis approach would be to project the gaze position vector (from central fixation) onto the object position vector. This approach yields high values during object fixation, low values elsewhere, and avoids opaque normalization steps (such as mean-centering across three items and normalizing by the mean distance) that rely on unreported values. I recognize that this could lead fixations past the eccentricity of the objects to have high values. I do think that treating fixations outside of a certain radius the same way they treat blinks would handle this sufficiently.

*Figure 2a: The right y-axis of Figure 2a still lacks a descriptive text label. It currently only lists units (DVA). However, because the text states this metric undergoes normalization, it should theoretically be expressed in arbitrary units (a.u.). Please clarify or correct this axis.

*Data Reporting: Throughout the manuscript, the authors should report raw accuracy values (and chance-corrected accuracy where appropriate) rather than just reporting the significance of statistical tests. This will greatly improve the transparency and informativeness of the results

Donk M, van Heusden E, Olivers CNL. Retinal eccentricity modulates saliency-driven but not relevance-driven visual selection. Atten Percept Psychophys. 2024 Jul;86(5):1609-1620. doi: 10.3758/s13414-024-02848-z. Epub 2024 Jan 25. PMID: 38273181; PMCID: PMC11557663.

Reviewer #3: The authors have carefully addressed all the concerns raised in the first round of reviews. I have no further concerns.

# Author comment: Author response to Decision Letter 2

**DOI:** 10.1371/journal.pbio.3004047.r005

21 Sep 2026

Attachment Submitted filename: response to review_2nd.docx

# Editor report: Decision Letter 2

**DOI:** 10.1371/journal.pbio.3004047.r006

**Authors:** Lucas Smith

24 Sep 2026

Dear Dr Huang,

Thank you for the submission of your revised Research Article "Adaptive sequential eye-movement sampling and reorganization under different task demands" for publication in PLOS Biology and thank you for addressing the last reviewer and editorial requests in this revision. On behalf of my colleagues and the Academic Editor, Christopher Pack, I am pleased to say that we can in principle accept your manuscript for publication, provided you address any remaining formatting and reporting issues. These will be detailed in an email you should receive within 2-3 business days from our colleagues in the journal operations team; no action is required from you until then. Please note that we will not be able to formally accept your manuscript and schedule it for publication until you have completed any requested changes.

**IMPORTANT - as you address any production requests, to come, we also ask that you address the following editorial requests.

1) TITLE: After some discussion within the team, we would like to suggest a tweak to the title to make it more active. If you agree (and if supported), we suggest you change the title to:

"Eye movements reflect capacity-dependent organization of working memory"

^we are happy to discuss this further if you wish.

2) ETHICS STATEMENT: Thank you for confirming in your response to my requests that your study was conducted in accordance with the principles expressed in the Declaration of Helsinki. I did not see this statement added into your manuscript. Can you make sure you add that statement to your methods section?

3) DATA: Thank you for citing your OSF deposition with underlying data in your figure legends. Please add a similar sentence, pointing readers to the underlying data, in the supplemental figure legends as well.

Please take a minute to log into Editorial Manager at http://www.editorialmanager.com/pbiology/, click the "Update My Information" link at the top of the page, and update your user information to ensure an efficient production process.

PRESS

We frequently collaborate with press offices. If your institution or institutions have a press office, please notify them about your upcoming paper at this point, to enable them to help maximise its impact. If the press office is planning to promote your findings, we would be grateful if they could coordinate with biologypress@plos.org. If you have previously opted in to the early version process, we ask that you notify us immediately of any press plans so that we may opt out on your behalf.

We also ask that you take this opportunity to read our Embargo Policy regarding the discussion, promotion and media coverage of work that is yet to be published by PLOS. As your manuscript is not yet published, it is bound by the conditions of our Embargo Policy. Please be aware that this policy is in place both to ensure that any press coverage of your article is fully substantiated and to provide a direct link between such coverage and the published work. For full details of our Embargo Policy, please visit http://www.plos.org/about/media-inquiries/embargo-policy/.

Thank you again for choosing PLOS Biology for publication and supporting Open Access publishing. We look forward to publishing your study.

Sincerely,

Luke

Lucas Smith, Ph.D.

Senior Editor

PLOS Biology

lsmith@plos.org
